# The microbiota continuum along the female reproductive tract and its relation to uterine-related diseases

**DOI:** 10.1038/s41467-017-00901-0

**Published:** 2017-10-17

**Authors:** Chen Chen, Xiaolei Song, Weixia Wei, Huanzi Zhong, Juanjuan Dai, Zhou Lan, Fei Li, Xinlei Yu, Qiang Feng, Zirong Wang, Hailiang Xie, Xiaomin Chen, Chunwei Zeng, Bo Wen, Liping Zeng, Hui Du, Huiru Tang, Changlu Xu, Yan Xia, Huihua Xia, Huanming Yang, Jian Wang, Jun Wang, Lise Madsen, Susanne Brix, Karsten Kristiansen, Xun Xu, Junhua Li, Ruifang Wu, Huijue Jia

**Affiliations:** 1 BGI-Shenzhen, Shenzhen, 518083 China; 2 China National Genebank, BGI-Shenzhen, Shenzhen, 518120 China; 3 BGI Education Center, University of Chinese Academy of Sciences, Shenzhen, 518083 China; 4 Peking University Shenzhen Hospital, Shenzhen, 518036 China; 5 Shenzhen Key Laboratory on Technology for Early Diagnosis of Major Gynecological diseases, Shenzhen, 518036 China; 6 Department of Biology, Laboratory of Genomics and Molecular Biomedicine, University of Copenhagen, Universitetsparken 13, 2100 Copenhagen, Denmark; 7 Shenzhen Engineering Laboratory of Detection and Intervention of human intestinal microbiome, BGI-Shenzhen, Shenzhen, 518083 China; 8 Qingdao University-BGI Joint Innovation College, Qingdao University, Qingdao, 266071 China; 9 Shenzhen Key Laboratory of Human Commensal Microorganisms and Health Research, BGI-Shenzhen, Shenzhen, 518083 China; 10 James D. Watson Institute of Genome Sciences, Hangzhou, 310000 China; 11 Macau University of Science and Technology, Taipa, Macau, 999078 China; 12 National Institute of Nutrition and Seafood Research, (NIFES), Postboks 2029, Nordnes, N-5817 Bergen, Norway; 13 Department of Biotechnology and Biomedicine, Technical University of Denmark, Soltofts Plads, Building 221, 2800 Kongens Lyngby, Denmark; 14 School of Bioscience and Biotechnology, South China University of Technology, Guangzhou, 510006 China

## Abstract

Reports on bacteria detected in maternal fluids during pregnancy are typically associated with adverse consequences, and whether the female reproductive tract harbours distinct microbial communities beyond the vagina has been a matter of debate. Here we systematically sample the microbiota within the female reproductive tract in 110 women of reproductive age, and examine the nature of colonisation by 16S rRNA gene amplicon sequencing and cultivation. We find distinct microbial communities in cervical canal, uterus, fallopian tubes and peritoneal fluid, differing from that of the vagina. The results reflect a microbiota continuum along the female reproductive tract, indicative of a non-sterile environment. We also identify microbial taxa and potential functions that correlate with the menstrual cycle or are over-represented in subjects with adenomyosis or infertility due to endometriosis. The study provides insight into the nature of the vagino-uterine microbiome, and suggests that surveying the vaginal or cervical microbiota might be useful for detection of common diseases in the upper reproductive tract.

## Abstract (web summary)

Whether the female reproductive tract harbours distinct microbiomes beyond the vagina has been a matter of debate. Here, the authors show a subject-specific continuity in microbial communities at six sites along the female reproductive tract, indicative of a non-sterile environment.

## Introduction

In marsupials and placental mammals, the female reproductive tract has developed unique structures such as the vagina and the uterus. During the reproductive cycle, mature oocytes from the ovaries enter the peritoneal cavity to be captured by fimbriae of the fallopian tubes. The oocytes are fertilised in the fallopian tubes, and the zygotes develop and translocate to the uterus for implantation. While the vagina is home for trillions of bacteria, the uterus and the fallopian tubes are generally believed to be sterile, which would require the cervix to be a perfect barrier. Mucins in the cervix, however, are known to change conformation, leading to aggregations dependent on pH variations during the menstrual cycle^1^. Such changes may in theory allow passage of bacteria during certain conditions.

*Lactobacilli* are known as the keystone species of the vaginal microbiota in reproductive-age women^2^. Indeed, culture-independent 16S rRNA gene amplicon sequencing studies from the United States have identified 5 community types of vaginal microbiota, 3 or 4 of which contain >90% *Lactobacillus* ^3–5^. The lactic acid produced by the vaginal microbiota helps maintain a low pH of 3.5–4.5, a major factor in limiting the growth of potentially harmful bacteria. Alterations in the vaginal microbiota play a role in common conditions such as bacterial vaginosis, sexually transmitted diseases, urinary infections and preterm birth^2, 6, 7^.

In contrast, the upper reproductive tract remains largely unexplored. Bacteria have mostly been studied in small sample sizes in the context of infection, especially in relation to preterm birth^6^. Although controversial, the placenta has recently been reported to harbour a microbiota^8^. It is as yet not clear what type of bacteria, if any, may exist in the upper reproductive tract of the vast majority of women who are not in periods of infection or pregnancy. Furthermore, it is not known if an upper reproductive tract microbiota could play a role in uterine-related diseases such as hysteromyoma, adenomyosis and endometriosis.

Here, we systematically sampled the microbiota at six sites within the female reproductive tract, from a large cohort of Chinese women of reproductive age. Bacteria were identified by using 16S rRNA gene amplicon sequencing, real-time qPCR, as well as conventional bacterial culturing. The results indicate a continuity of the vagino-uterine microbiome, with a distinct trend within the same individual. Potential bacterial markers for adenomyosis and endometriosis were also identified.

## Results

### Microbiota composition at six sites within the female reproductive tract

**Fig. 1:**
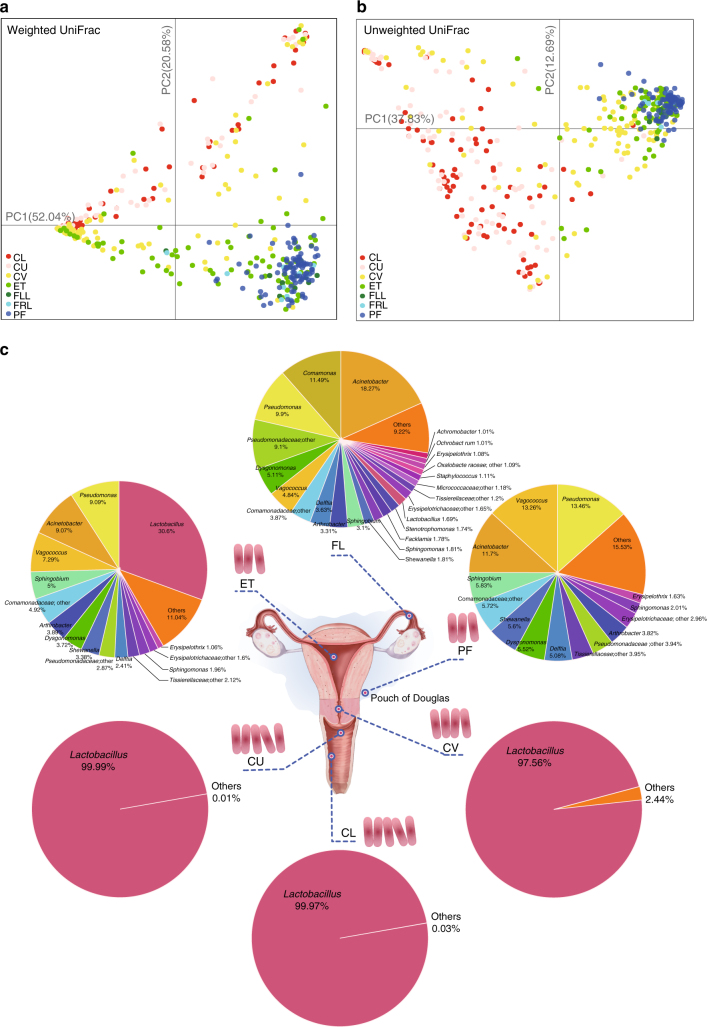
Composition of the vagino-uterine microbiota. **a**, **b** PCoA on the samples based on weighted (**a**) and unweighted (**b**) UniFrac distances. Samples were taken from CL, CU and CV before operation, and from ET, FLL, FRL and PF during operation. Each dot represents one sample. **c** Pie chart for the microbial genera at each body site according to the median relative abundance. Genera that took up <1% of the microbiota were labelled together as ‘others’. A pink rod represents about 10^2^ copies/sample, according to the qPCR results in Supplementary Fig. 5e. Samples derive from the study cohort of 95 reproductive-age women (*n* = 94 CL, 95 CU, 95 CV, 80 ET, 93 PF, 9 FLL and 10 FRL, Supplementary Data 1)

To explore the microbiota beyond the vagina, we collected samples from six locations (CL, lower third of vagina; CU, posterior fornix; CV, cervical mucus drawn from the cervical canal; ET, endometrium; FLL and FRL, left and right fallopian tubes; PF, peritoneal fluid from the pouch of Douglas) throughout the female reproductive tract from an initial study cohort of 95 Chinese women submitted to surgery for conditions not known to involve infections (Fig. 1). These conditions included hysteromyoma (also known as uterine fibroids), adenomyosis, endometriosis and salpingemphraxis, which are to our knowledge the best proxies for the upper reproductive tract in healthy young women (Supplementary Data 1). Samples from the vagina and the cervical mucus (CL, CU, CV) were taken upon visit to the clinic (without any prior disturbance). Samples from the peritoneal and uterine sites (PF, FL, ET) were taken during laparoscopy or laparotomy from minimally invasive surgery cuts to avoid possible contamination from the vaginal microbiota if the samples were to go through the cervical os (Fig. 1, Supplementary Fig. 1). The samples were subjected to 16S rRNA gene amplicon sequencing, quality-controlled and clustered into operational taxonomic units (OTUs) (Supplementary Data 2, 3).

The lower (CL, CU, CV) and upper (ET, FL, PF) reproductive tract samples separated when subjected to principal coordinate analyses (PCoA) based on weighted and unweighted UniFrac distances (Fig. 1a, b). The FLL and FRL showed essentially the same microbiota (Fig. 1a, b), and were therefore clustered into a common FL category for further analysis (except for calculation of intra-individual and inter-individual differences, referred to later).

Consistent with previous reports^3–5^, the CL as well as the CU were dominated by the *Lactobacillus* genus (Fig. 1c), and exhibited a low α-diversity (Supplementary Fig. 2a). At the species level, the CL and CU samples contained *L. crispatus*, *L. iners* and other *Lactobacillus* spp. (Supplementary Fig. 2b), similar to previous reports from the US^3–5^. Notably, CV samples generally contained a lower proportion of *Lactobacillus* than the vaginal samples (Fig. 1c), and varied among the sampled individuals (Supplementary Fig. 3a). In the ET samples, *Lactobacillus* no longer dominated, and bacteria such as *Pseudomonas*, *Acinetobacter*, *Vagococcus* and *Sphingobium* constituted a notable fraction of the microbiota (Fig. 1c, Supplementary Fig. 3b). The proportion of these bacteria further increased at the openings of the FL, leaving a median relative abundance of 1.69% for *Lactobacillus* (Fig. 1c, Supplementary Fig. 3d). The PF samples generally lacked the presence of *Lactobacillus*, but otherwise harboured a microbiota as diverse but not completely the same as the FL samples (Fig. 1, Supplementary Fig. 3c).

At the phylum level, the Firmicutes-dominated lower reproductive tract microbiota contrasted the large proportions of Proteobacteria, Actinobacteria and Bacteroidetes in the upper reproductive tract (Supplementary Fig. 3e).

As noted above, we took samples from the uterine and peritoneal sites without going through the cervical os to address a major concern in the field that transcervical samples might be contaminated by the vaginal microbiota. We nonetheless tested the specificity of sampling route by collection of ET samples via the uterus as well as through the cervical os, and of CV from the cervical os as well as from the uterine end. The distribution of bacteria in the samples taken through the cervical os showed high similarity to that in samples taken by opening the uterus during surgery (Supplementary Fig. 4a, b), suggesting that both the uterine and cervical microbiota could be readily accessed and analysed in women not undergoing surgery.

### Estimation of bacterial biomass in the female reproductive tract

In order to provide some absolute measure of the microbiota beyond the vagina, we developed a species-specific real-time qPCR approach, focusing on the dominant vaginal bacteria *Lactobacilli*. The abundances of *L. crispatus*, *L. iners*, *L. gasseri* and *L. jensenii* monotonously decreased from CL, CU, CV, to ET and PF (Supplementary Fig. 5a–d), consistent with genus level data from 16S rRNA gene amplicon sequencing (Fig. 1). The concordance between qPCR and amplicon sequencing results were further confirmed by Spearman’s correlation, which showed a correlation coefficient of 0.72 for *L. crispatus* and 0.56 for *L. iners*, the two dominant *Lactobacilli* species (Supplementary Fig. 2b).

The total bacterial biomass within each site was then calculated based on the copy number from qPCR, divided by the corresponding relative abundance in the sample according to 16S rRNA gene sequencing. This calculation revealed that, while the vaginal sites contained about four orders of magnitude more bacteria (10^10^–10^11^), the PF samples contained similar bacterial biomass as the ET samples (Supplementary Fig. 5e), which are orders of magnitude above potential background noise^9^. The negative controls (sterile phosphate-buffered saline (PBS), sterile saline and ultrapure water) showed much higher cycle threshold (Ct values) than the low biomass ET and PF samples (Supplementary Table 1, Supplementary Fig. 1), signifying that much less bacterial DNA was detected in non-bio control samples. It is thus important to bear in mind that the higher bacterial diversity in the upper reproductive tract corresponds to a lower bacterial biomass than the much better established vaginal microbiota (Fig. 1c).

### Cultivation of live bacteria from the upper reproductive tract

Another important question is whether live bacteria rather than debris makes up the bacterial DNA signal in the upper reproductive tract samples. Hence, in a validation study we performed 16S rRNA gene amplicon sequencing along with culturing of live bacteria from an additional cohort of 15 women.

Of the 21 diluent negative controls, 19 had lower DNA concentrations and library concentrations (close to zero) than their respective tissue samples (Supplementary Data 4), again confirming that there was little DNA in the non-bio control samples. In PCoA, microbial profiles differed between the low biomass upper reproductive tract samples and diluent controls (Supplementary Fig. 6, sterile saline for the peritoneal samples and PBS for the remaining samples).

The same samples extracted at different times, diluted or undiluted before amplification, or sequenced in different runs showed high reproducibility, indicating that random contamination had no major impact on bacterial community structure detected in the upper reproductive tract (Supplementary Table 2, Supplementary Data 5).

We further attempted to culture and isolate live bacteria from the freshly collected samples. PF samples were plated on peptone yeast extract-glucose (PYG) agar with 5% horse blood and incubated aerobically or anaerobically. Positive bacterial cultures were obtained from 5 out of 15 subjects, resulting in 8 different isolates belonging to 7 genera such as *Lactobacillus*, *Staphylococcus* and *Actinomyces* (Supplementary Table 3). These genera were also detected in our 16S rRNA gene amplicon sequencing data, and supported by previous cultivation of vaginal or amniotic samples^10–16^ (Supplementary Table 3). Reassuringly, no bacteria were isolated from the diluent-negative controls (including swabs from sterilised skin samples from the patients and the gloves used by doctors). Thus, live bacteria do exist in the upper reproductive tract, of which we succeeded in isolation of some using conventional culturing methods.

### Signature species for each site of the reproductive tract

**Fig. 2:**
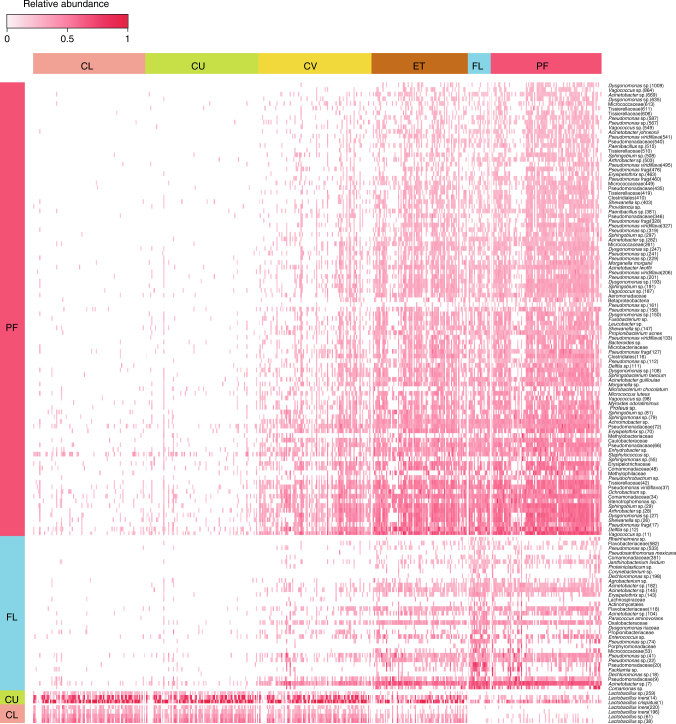
Signature species for each body site. Heatmap for the relative abundances of signature OTUs from each body site (*P* < 0.05, multi-level pattern analysis, and IndVal >0.5). Each bar represents one sample (*n* = 94 CL, 95 CU, 95 CV, 80 ET, 93 PF, 9 FLL and 10 FRL, Supplementary Data 1)

To get more insight into the ecology of the vagino-uterine communities, we defined signature OTUs for each body site considering both the occurrence and abundance of the given OTUs (*P* < 0.05 in multi-level pattern analysis, and indicator value index (IndVal) >0.5, Fig. 2, Supplementary Data 6). *L. iners* and *L. crispatus* were identified as signature species of the vaginal sites (CL and CU). No signature OTUs with IndVal >0.5 was identified for CV or ET, suggesting that they are bacterial transition zones between the vagina and the upper anatomical sites. The FL had a number of signature OTUs including *Pseudomonas*, *Erysipelothrix* and *Facklamia* (Fig. 2, Supplementary Data 6). The PF featured many signature OTUs, including *Pseudomonas*, *Morganella*, *Sphingobium* and *Vagococcus*, all of which increased monotonously from the CU and the CV to the ET and PF (Supplementary Data 6). In support of the existence of these taxa in the upper reproductive tract, they have been reported in previous studies of the vaginal microbiota in humans or animals^17–21^. Overall, we observed the female reproductive tract as a habitat for facultative anaerobes or aerobes; the upper sites contained a variety of bacteria that grow in mildly alkaline conditions, contrasting the *Lactobacillus*-dominated low pH environment of the vagina.

### Intra-individual and inter-individual similarity in the vagino-uterine microbiota

**Fig. 3:**
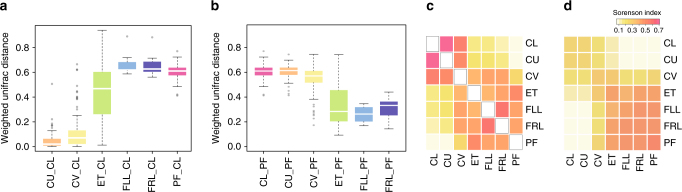
Similarity of the vagino-uterine microbiota within and between individuals. **a**, **b** Weighted UniFrac distance of the microbiota at each body site relative to the CL (**a**) and PF (**b**) in the same individual. Boxes denote the interquartile range (IQR) between the first and third quartiles (25th and 75th percentiles, respectively), and the line inside the boxes denote the median. The whiskers denote the lowest and highest values within 1.5 times the IQR from the first and third quartiles, respectively. Circles represent data points beyond the whiskers. **c** Intra-individual Sorenson indices between different body sites. **d** Inter-individual Sorenson indices between different body sites. Samples derive from the study cohort of 95 reproductive-age women (*n* = 94 CL, 95 CU, 95 CV, 80 ET, 93 PF, 9 FLL and 10 FRL, Supplementary Data 1)

To examine the microbiota relationship at the six sites of the reproductive tract, we computed the intra-individual and inter-individual distances between samples from each individual. Weighted intra-individual UniFrac distance relative to CL samples gradually increased from CU and CV to ET and PF (Fig. 3a), consistent with the anatomical contingency of these sites (Fig. 1). The same was also true in reverse order, i.e. weighted intra-individual UniFrac distance relative to PF gradually increased from ET, CV, to CU and CL (Fig. 3b). Samples from the same individual were highly correlated, and the Sorenson indices between body sites were consistent with their anatomical contingency (Fig. 3c). The intra-individual correlation was notable even between the less invasive sampling site of CV and the less reachable PF (median of Sorenson index = 0.255, Fig. 3c), hinting at the possibility of minimally invasive surveys of the uterine and peritoneal microbiota by cervical mucus sampling in the general population.

Of note, the clear distinction between the upper and lower reproductive tract, intersecting at CV and ET, remained intact even when performing an inter-individual comparison (Fig. 3d) (although the level of the inter-individual indices diminished from that of the intra-individual (Fig. 3c)), hence underscoring both anatomical as well as individual-specific features of the vagino-uterine microbiota.

### Site-specific community types in the vagino-uterine microbiota

**Fig. 4:**
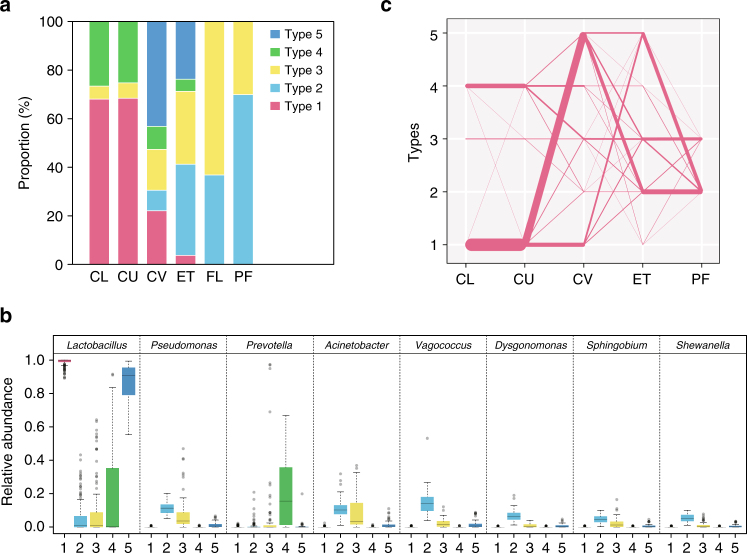
Community types of the vagino-uterine microbiota. **a** Distribution of samples among the five community types at each body site (*n* = 94 CL, 95 CU, 95 CV, 80 ET, 93 PF, 9 FLL and 10 FRL, Supplementary Data 1). **b** Relative abundances of the top 8 most abundant genera in the 5 community types. The boxes denote the interquartile range (IQR) between the first and third quartiles (25th and 75th percentiles, respectively), and the line inside the boxes denote the median. The whiskers denote the lowest and highest values within 1.5 times the IQR from the first and third quartiles, respectively. Circles represent data points beyond the whiskers. **c** Community types connected between neighbouring sites according to individual subjects. Due to the small sample size, results for the fallopian tubes were plotted in a separate graph (Supplementary Fig. 7c). The width of each line indicates the number of subjects (*n* = 1–95)

To further understand the relationship between communities at each site, we examined community types in all samples using the Dirichlet multinomial mixture (DMM) model^5^. Of the five community types detected by DMM (Supplementary Fig. 7), Type 1 and 5 were dominated by the *Lactobacillus* genus, which was present in notable amounts also in Type 2, 3 and 4 (Fig. 4a, b, Supplementary Data 7). Type 4, which contained a higher proportion of *Prevotella* and might correspond to the more diverse vaginal microbiota type reported previously^3–5^, was detected in some of the CV and ET samples but was not found in the FL and PF samples of this cohort (Fig. 4, Supplementary Fig. 7). The major type in PF, Type 2 was also present in CV, while Type 3 was present at all sites, yet dominating in the FL samples (Fig. 4, Supplementary Fig. 7).

When we examined transitions between community types at neighbouring sites, most subjects with Type 1 in the vagina (CL) remained Type 1 at the CU, and changed to Type 5 either at CV or at ET, and then turned to Type 2 in PF (Fig. 4c). By contrast, subjects with Type 3 or 4 at the vaginal sites rarely became Type 5 at the uterine sites, while some Type 4 subjects were found to switch to Type 3 in the upper part (Fig. 4b, Supplementary Fig. 7c). These findings corroborated our aforementioned results that there is an intra-individual continuum of the microbiota from the vagina to the peritoneal fluid that gradually changes according to the habitat.

### Lifestyle and clinical factors associated with microbiota changes

The vaginal microbiota has previously been reported to vary between ethnic groups (in the US) and the menstrual cycle^3, 4^. With our comprehensive collection of metadata (Supplementary Data 1), we examined whether various clinical and lifestyle factors were associated with changes in the vagino-uterine microbiota in women of reproductive age. Factors such as age of initial sexual intercourse, duration of menstrual period, and gravida and para appeared associated with microbiota composition at one or more sites, but failed to reach statistical significance after controlling for multiple testing (permutational multivariate analysis of variance (PERMANOVA), *P* < 0.05 but *q* > 0.05, Supplementary Data 8 and 9). Having given birth (multipara versus nullipara) was associated with microbiota composition in CV and PF (*q* < 0.05, Supplementary Data 9). Herbal medication was associated with microbiota composition in the CV only (*q* < 0.05, Supplementary Data 9). Age, hysteromyoma, adenomyosis and endometrosis were also associated with microbiota composition in the PF (*q* < 0.05, Supplementary Data 8, 9). Therefore, variations in the composition of the vagino-uterine microbiota are associated with a number of natural and diseased physiological conditions important for women’s health.

### Identification of diseases based on the vagino-uterine microbiota

We further examined whether variations in the vagino-uterine microbiota composition were associated with different physiological conditions and diseases of the reproductive tract.

In relation to hysteromyoma (benign tumours in the uterus), *Lactobacillus* sp. were found to be more abundant in the vaginal and cervical samples of individuals with no hysteromyoma, while *L. iners* was more abundant in the CV of individuals with hysteromyoma (Supplementary Fig. 8). Among the hysteromyoma-enriched OTUs, one OTU in the CU and 11 OTUs in the PF were also identified to be influenced by the stage in the menstrual cycle (Spearman’s correlation coefficient >0.3 or <−0.3, *q* < 0.05, Supplementary Fig. 8); however, none of these OTUs showed a correlation with age. Overall, the results were consistent with hysteromyoma being associated with a minimally altered vagino-uterine microbiota.

Subjects with adenomyosis (abnormal presence of endometrial tissue within the myometrium) showed depletion or enrichment of many bacteria throughout the reproductive tract, some of which overlapped with bacteria associated with anaemia (Supplementary Figs. 9 and 10), which was consistent with clinical links between the two conditions (*P* = 0.001062 between the two conditions in our cohort, Fisher’s exact test). Furthermore, cross-validated random forest models distinguished subjects with adenomyosis from those without according to OTUs from any of the sites (Supplementary Fig. 11, Supplementary Data 10).

**Fig. 5:**
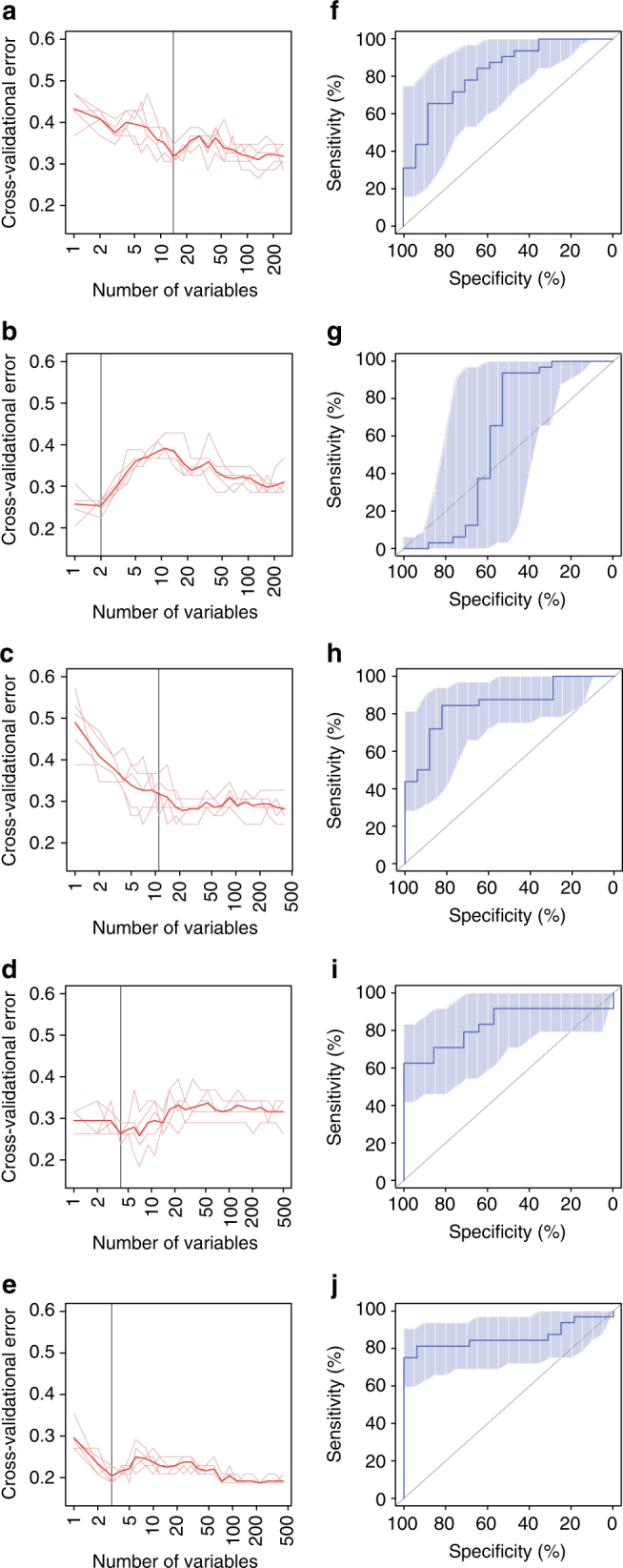
Microbiota-based classification of infertility due to endometriosis. **a**, **b**, **c**, **d**, **e** Distribution of 5 trials of 10-fold cross-validation error in random forest classification of fertile versus infertile samples as the number of OTUs increases (**a**, CL; **b**, CU; **c**, CV; **d**, ET; **e**, PF). The model was trained using relative abundance of the OTUs (present in at least 10% of the samples) in the samples (*n* = 16 without endometriosis (fertile), 32 with endometriosis (infertile)). Subjects with endometriosis who had given birth, and infertile subjects due to reasons other than endometriosis, e.g. salpingemphraxis, were not analysed (Supplementary Data 1). The red curve indicates an average of the five trials (pink lines). The grey line marks the number of OTUs in the optimal set. (**f**, **g**, **h**, **i**, **j**) Receiver operating curve (ROC) for the cross-validated sample set (**f**, CL; **g**, CU; **h**, CV; **i**, ET; **j**, PF). The area under receiver operating curve (*AUC*) is 0.8272, 0.5919, 0.8493, 0.8304 and 0.8613, respectively. The 95% confidence intervals (CI) are shown as shaded areas. The *diagonal lines* mark an AUC of 0.5 (i.e. random classification).

The microbiota-based models also allowed us to distinguish subjects without and with infertility attributed to endometriosis, a condition where endometrial cells present abnormal growth outside the uterus, and a major cause of infertility (Fig. 5, Supplementary Data 11). Altogether, these results suggest that the composition of the vagino-uterine microbiota potentially could be used to detect a number of ailments common to reproductive age women.

### Functional inferences of the reproductive tract microbiota

To estimate the functional capacities of the vagino-uterine microbiota, we predicted KOs (Kyoto Encyclopedia of Genes and Genomes (KEGG) Orthology groups) from the 16S rRNA gene data.

In the case of adenomyosis, the analyses revealed that adenomyosis affected functions throughout the vagino-uterine microbiota (Supplementary Fig. 12). The microbiota in subjects with adenomyosis were especially enriched in pathways involved in flagella assembly and biosynthesis of aromatic amino acids, while the microbiota in subjects without adenomyosis were enriched in phosphotransferase system and fatty acid biosynthesis. These results suggest that functionally, the microbiota is also a continuum in the reproductive tract, and could be perturbed at multiple sites in a disease.

### Menstrual cycle relates to the vagino-uterine microbiota and its function

**Fig. 6:**
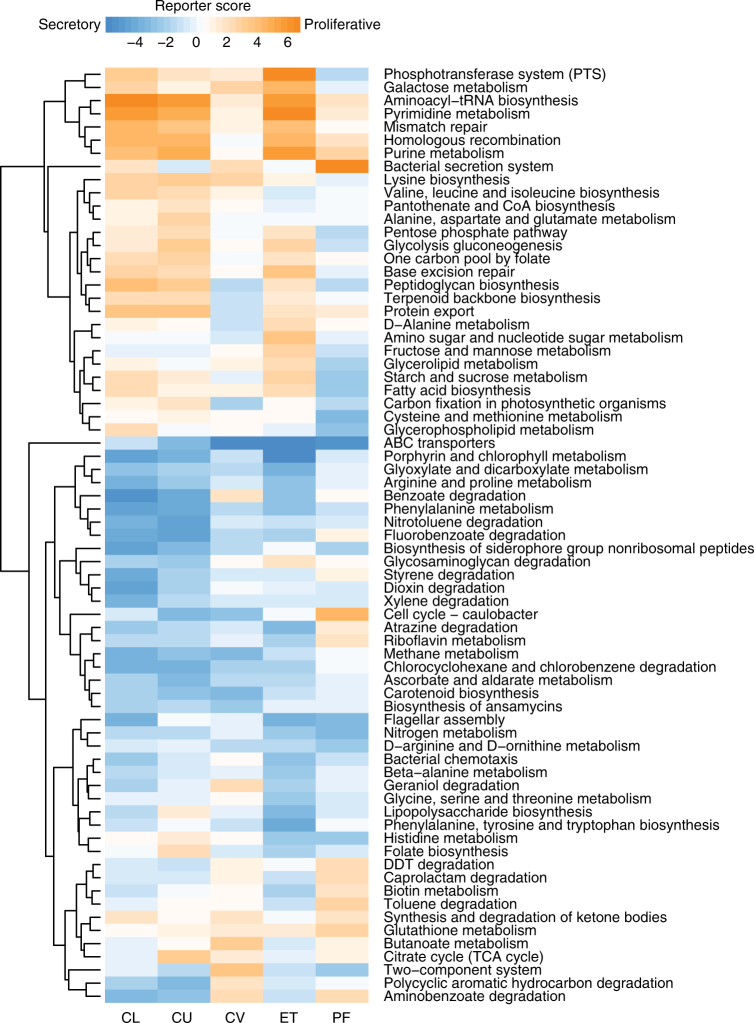
Associations between inferred microbial functions and menstrual phases. Microbial KEGG pathways enriched in the proliferative or the secretory phases (orange versus blue, reporter score >1.96 or <−1.96) and present in all the body sites were plotted as a heatmap. The pathways were arranged by unsupervised hierarchical clustering. Samples derive from the study cohort of 95 reproductive-age women (*n* = 94 CL, 95 CU, 95 CV, 80 ET, 93 PF, 9 FLL and 10 FRL, Supplementary Data 1)

Differences in the vagino-uterine microbiota were also identified between phases of the menstrual cycle, as was apparent from cross-validated random-forest models that classified samples into different phases of the cycle (Supplementary Fig. 13). OTUs that led to optimal classification included *L. iners*, and *Lactobacillus* sp. from vaginal sites, *Sphingobium* sp., *Propionibacterium acnes* and *Pseudomonas* sp. from the ET or PF, which were differentially enriched during the proliferative and secretory phases (Supplementary Fig. 13c). Notably, *P. acnes* was more abundant in the secretory phase in the ET (Supplementary Fig. 13l), and has previously been identified in the placenta and cultured from follicular fluid^8, 22–24^. Functionally, the proliferative phase appeared associated with increased bacterial proliferation in the vagina and ET compared to the secretory phase, seen as higher abundance of pathways for pyrimidine and purine metabolism, aminoacyl-tRNA biosynthesis, and peptidoglycan biosynthesis (Fig. 6). The secretory phase, on the other hand, showed higher abundance of pathways for porphyrin metabolism, arginine and proline metabolism, degradation of benzoate, nitrotoluene and biosynthesis of siderophore (Fig. 6). These results indicate that the vagino-uterine microbiota probably varies according to the menstrual cycle.

## Discussion

Herein we demonstrate the existence of distinct bacterial communities throughout the female reproductive tract forming a continuum of microbiotas changing from the vagina to the ovaries, which challenge the traditional view of human foetal development being a sterile event^8, 25–27^. Indeed, vertical transmission of the mothers’ microbiota before birth could be the norm throughout the animal kingdom, as previously alluded to by others^28^. Demonstrating a microbiota in traditionally unchartered territories remains a challenging task, and as illustrated here, the combination of high-throughput sequencing, quantitative PCR, culturing of live bacteria and other techniques would make a stronger case. Endosymbiotic, instead of free-living bacteria, is also an intriguing possibility.

As it is not possible to directly sample the upper reproductive tract (without going through the cervical os. and especially for FL, PF) of fully healthy women, we have included a handful of fertile and infertile conditions that are not known to involve infection. The relevance of the vagino-uterine microbiota to female fecundity would require further analyses in large cohorts and in model systems. Although differing by disease cohorts and methodology, our sequencing-based analyses nevertheless showed some similarity to an early study on chronic endometritis^29^ (Supplementary Table 4) and a recent study on endometrial cancer^30^ (Supplementary Table 5). Notably, the latter study also pointed to the exciting possibility of idendifying endometrial cancer through sampling the vaginal microbiota^30^. How disturbances to the vagino-uterine microbiota might eventually lead to benign or malignant conditions threatening the health of pre-menopausal and post-menopausal women is a major open question.

Of relevance for clinical practice was the finding of intra-individual correlations between microbiota of the PF and that of CV, as this indicates that (minimally invasive) sampling of cervical mucosa could be used to survey the status of the uterus and peritoneal cavity in the general population. This is of relevance in relation to the demonstrated associations between the uterine microbiota and diseases such as adenomyosis and endometrosis. We also showed that microbiota at the upper reproductive tract can be available through laparoscopy with minimal artificial effects and contaminations, laying the basis for the usage of this sampling route in subsequent studies.

We find that both the upper and the lower reproductive tract are home for facultative anaerobes and aerobes, in contrast to the situation in bacterial vaginosis (and more recently also reported in endometrial cancer^30^, Supplementary Table 5), represented by overgrowth with obligate anaerobes, being linked to adverse outcomes such as pre-term birth and sexually transmitted diseases^2, 6, 7^. Clothing, sedentary lifestyle, contraception and delayed pregnancies common in modern life may modulate the vagino-uterine microbiota. While vaginal *Lactobacillus* species are known to inhibit other bacteria by maintaining a high concentration of lactic acid and hydrogen peroxide production, a healthy uterine microbiota likely also depends on the nutrients and hormones available to the uterus, as well as the microbiota in the vagina and the peritoneal cavity^1, 31^. In this regard, it was striking to identify associations between inferred microbial function and uterine-related diseases as well as to the menstrual cycle, which would require validations in larger and prospective cohorts^32^.

## Methods

### Study cohort and sample collection

The study was approved by the institutional review boards at Peking University Shenzhen Hospital and BGI-Shenzhen. As an initial cohort, 95 reproductive age women operated for conditions not known to involve infection (hysteromyoma, adenomyosis, endometriosis, salpingemphraxis) were included in the study by Peking University Shenzhen Hospital between December 2013 and July 2014 (Supplementary Data 1). Subjects with vaginal inflammation, severe pelvic adhesion, any acute inflammation, cancer, endocrine or autoimmune disorders were excluded. The subjects had no recorded recent use of hormones, antibiotics and vaginal medications, no cervical treatment within a week, no douching within 5 days and no sexual activity within 48 h. None of the subjects were pregnant, lactating or in menses at the time of sampling. Informed consents were obtained from all participants.

Samples from the lower reproductive tract (CL, CU, CV) were taken on the day of the clinical visit with no prior perturbation. Samples from the upper reproductive tract (ET, FL, PF) were taken during operation (on average 2.81 days later, Supplementary Fig. 1, Supplementary Data 1). Laparoscopy was used except for subjects with adenomyosis who need laparotomy. Care was taken to avoid contamination by blood. PF was sampled at the Pouch of Douglas after sterile saline was injected into peritoneal cavity, and then the FL and ET were sampled upon opening.

Nylon flocked swabs (Chenyang Global Group, CY-93050 and CY-98000) were used for all samples except PF. The swab heads were then placed in sterile PBS and flash-frozen with liquid nitrogen, stored at −80 °C and transported on dry ice to BGI-Shenzhen.

### DNA extraction and 16S rRNA amplicon sequencing

DNA extraction was performed as described^33^. Sample was treated with Lysozyme, proteinase K and SDS, then purified by phenol-chloroform-isoamylalcohol, precipitated by glycogen, sodium acetate and cold isopropanol, washed with 70% ethanol and resuspended in 1 × TE buffer. The V4–V5 region of the 16S rRNA genes was amplified by polymerase chain reaction (PCR) with a universal forward primer and a unique barcoded fusion reverse primer (V4-515F: 5ʹ-GTGCCAGCMGCCGCGGTAA-3ʹ and V5-907R: 5ʹ- CCGTCAATTCMTTTRAGT-3ʹ, where M indicates A or C and R indicates purine).

PCR was performed using the following conditions: 3 min denaturation at 94 °C; 25 cycles of denaturation at 94 °C for 45 s, annealing at 50 °C for 60 s, elongation at 72 °C for 90 s; and final extension at 72 °C for 10 min. The amplicons were purified using the AMPure beads (Axygen). Barcoded libraries were generated by emulsion PCR, and sequenced in a V5 to V4 reverse direction on a 318 chip using the 400 bp sequencing kit of the Ion Torrent Personal Genome Machine (PGM) system according to manufacturer’s instructions.

### Processing of 16S sequencing data

The raw data from the PGM system were exported and pre-processed by using Mothur (V1.33.3)^34^. The criteria for high-quality reads included: (1) longer than 200 bp; (2) less than two mismatches with the degenerate PCR primers; (3) average quality score <25. OTUs based on the 16S rRNA gene sequences were generated with the identity cutoff of 97% using QIIME’s UCLUST program^35^. The seed sequences of each OTU were chosen for taxonomic classification against the Greengene reference sequences (gg_13_8_otus) by the UCLUST taxonomy assigner. The α-diversities and β-diversities, Unifrac analyses were also calculated in QIIME with taxonomic abundance profiles at the OTU or genus level.

For OTU #1 from our samples (an abundant OTU from the vagina), the seed sequence showed over 97% identity with the V4V5 region of both the *Lactobacillus kitasatonis* and *Lactobacillus crispatus* 16S rRNA genes. To resolve this ambiguity, full-length 16S rRNA gene and a 289 bp fragment of the conserved single-copy gene encoding phenylalanine-tRNA ligase beta subunit (PF03147) were cloned from a sample containing 99% OTU #1 (C056CU), and Sanger sequencing (Applied Biosystems 3730 DNA analyzer) confirmed both genes to be from *L. crispatus ST1* (NCBI gene ID: 9107847 and 9107287, respectively).

### Identification of signature OTUs

Signature OTUs were identified according to their IndVal values, which consider both the occurrence and abundance of a taxon^27, 36^:

$${\rm{IndVa}}{{\rm{l}}_{\rm{p}}} = \frac{{{a_{\rm{p}}}}}{a} \times \frac{{{n_{\rm{p}}}}}{{{N_{\rm{p}}}}}$$

where *a*_p_ is the sum of the abundances of species within the samples from body site p, *n*_p_ is the number of occurrences of the species within the samples from site p, *a* is the sum of the abundances of species from all body sites, *N*_p_ is the number of samples from site p.

Statistical tests on the IndVal values were performed as described previously, through permutation of the relative abundance of each genus or OTU across communities, using the multipatt function of the R package 'indicspeces'^27^.

### Concordance of the microbiota between samples

We used the Sorenson index (Sørensen–Dice index) to measure the similarity between microbiota at different body sites of the same individual, based on the presence and absence of OTUs. The index was calculated as:

$${\rm{QS}}\, = \,\frac{{2C}}{{A + B}} = \frac{{2\left| {A\mathop { \cap }\nolimits B} \right|}}{{\left| A \right| + \left| B \right|}}$$

where *A* and *B* are the number of OTUs in two samples A and B, respectively, and *C* is the number of OTUs shared by the two samples; QS is the quotient of similarity, which ranges from 0 to 1.

### Community types of the vagino-uterine microbiota

Relative abundances of genera in all 476 samples were used to determine the optimal number of clusters according to DMM^5^. Transitions between community types were assessed statistically from community type assignments for all subjects together, as well as for those who had fallopian tube samples.

### PERMANOVA on the influence of phenotypes

We applied PERMANOVA on the relative abundances of OTUs or metabolites in the samples to assess impact from each of the factors listed^37, 38^. We used Bray–Curtis or UniFrac distance and 9999 permutations in R (3.0.0, vegan package^38, 39^).

### Random-forest classification

In order to construct a model that could distinguish samples of different menstrual phases for each body site, the relative abundances of OTUs (found in at least 10% of the samples) in the samples were fitted against the samples’ actual days in the menstrual cycle using default parameters in the randomForest package in R (3.1.2 RC) except that 1000 trees were used, as in previous reports on the gut microbiota age of infants^27, 40^. Ten-fold cross-validation was performed five times. The cross-validation error curves from the five trials were averaged, and the minimum error in the averaged curve plus the standard deviation at that point were used as the cutoff for acceptable error. From the sets of OTUs with a classification error less than the cutoff, the set with the smallest number of OTUs was chosen as the optimal set^38, 41^.

Random-forest classification of samples according to non-continuous phenotypes was performed in the same manner.

MetaScope in the Progenesis QI software was used to search for compound identifications not only based on neutral mass, isotope distribution and retention time, but also based on collisional cross-sectional area and MS/MS fragmentation data in the HMDB database.

### Functional inference of 16S data

PICRUSt (phylogenetic investigation of communities by reconstruction of unobserved states) was used to infer KOs from OTU data as previously described^42^. The relative abundance of KEGG pathways and modules was summed from the relative abundance of KOs belonging to these pathways and modules.

Differentially enriched KEGG pathways or modules were identified according to their reporter score^27, 38, 43^, from the *Z*-scores of individual KEGG orthology groups. A reporter score of *Z* = 1.96 was used as a detection threshold for pathways or modules that were significantly over-represented in one group relative to the other, corresponding to 95% confidence.

### Real-time quantitative PCR

Four major vaginal *Lactobacillus* species including *L. crispatus*, *L.iners*, *L jensenii* and *L. gasseri* were quantified by real-time qPCR modified from a previously described regimen^44^. After DNA quantification was conducted for each sample using Qubit Fluorometer (Life Technologies), 1.6 μl DNA solution was used as the template for real-time PCR. Standard curves were generated using serial tenfold dilutions of plasmids. The range of amplification efficiency for the qPCR was from 90 to 110%, and linearity values were all ≥0.99. Real-time qPCR was conducted using SYBR Premix Ex Taq GC (TAKARA) using the StepOnePlus Real-time PCR System (Life Technologies). Each reaction included a standard curve, and each sample was run in triplicate and contained 10 μl SYBR Premix Ex Taq GC, 1.6 μl of DNA template along with 0.2 μM primers in a final reaction volume of 20 μl. Sterile PBS and sterile physiological saline were used as diluent negative controls, which were subject to sample processing, DNA extraction and real-time qPCR in parallel with samples according to our standard protocol, in order to determine the source of contamination. In addition, non-template control, which was ultrapure water, was also included when performing real-time qPCR. Total DNA amount for each sample was calculated by multiplying the concentration of DNA by the total volume of extracted DNA.

### Validation cohort used for control of contamination and bacterial culturing

We used an independent validation cohort comprised of additional 15 reproductive age women included between May 2016 and June 2016 (Supplementary Data 1). The hospital and conditions for surgery were the same as those in study cohort. Informed consents were also obtained from all participants.

Samples from the upper reproductive tract (ET, FL, PF) were collected during operation. For each operation, sterile PBS, sterile physiological saline, dry sterile swabs rubbed on patient's preoperative skin area, dry sterile swabs rubbed on surgeon's gloved fingers were sampled as diluent negative controls. The swab heads were then placed in sterile PBS (Supplementary Fig. 1).

PF samples and diluent negative controls from the additional 15 women were collected for cultivation experiment. All the samples were kept under anaerobic conditions, and processed in the laboratory within 6 h of sample collection to maximise recovery of bacterial viability. In total, 100 μl of peritoneal fluid specimens and 100 μl of each diluent negative controls were plated on PYG agar (DSMZ 104 medium) with 5% horse blood and incubated aerobically or anaerobically at 37 °C for up to 72 h. Reducing agents such as resazurin and cysteine-HCl were not added to cultures incubated anaerobically. Bacterial isolates from the plates were selected based on morphologic characteristics. DNA from single colonies was extracted using the Bacterial DNA Kit (OMEGA) and then amplified using the universal primers 27F/1492R^45^. The amplicons were purified using the Magbind PCR Purification Kit (GeneOn BioTeach) and then sent for Sanger sequencing. The sequences were analysed using the GenBank 16S rRNA sequences database using the BLAST algorithm, and sequences with more than 97% similarity were considered to be of the same species.

### Data availability

Sequence reads for the 544 samples have been deposited in the European Nucleotide Archive under study numbers PRJEB16013 and PRJEB21098. The authors declare that all other relevant data supporting the findings of the study are available in this article and its Supplementary Information files, or from the corresponding author upon request.

## Electronic supplementary material

Supplementary Information

Description of Additional Supplementary Files

Supplementary Data 1

Supplementary Data 2

Supplementary Data 3

Supplementary Data 4

Supplementary Data 5

Supplementary Data 6

Supplementary Data 7

Supplementary Data 8

Supplementary Data 9

Supplementary Data 10

Supplementary Data 11

## References

[1] Brunelli R (2007). Globular structure of human ovulatory cervical mucus. FASEB J..

[2] Ma B, Forney LJ, Ravel J (2012). Vaginal microbiome: rethinking health and disease. Annu. Rev. Microbiol..

[3] Ravel J (2010). Vaginal microbiome of reproductive-age women. Proc. Natl Acad. Sci. USA.

[4] Gajer P (2012). Temporal dynamics of the human vaginal microbiota. Sci. Transl. Med..

[5] Ding T, Schloss PD (2014). Dynamics and associations of microbial community types across the human body. Nature.

[6] Goldenberg RL, Hauth JC, Andrews WW (2000). Intrauterine infection and preterm delivery. N. Engl. J. Med..

[7] Hyman RW (2013). Diversity of the vaginal microbiome correlates with preterm birth. Reprod. Sci..

[8] Aagaard K (2014). The placenta harbors a unique microbiome. Sci. Transl. Med..

[9] Salter SJ (2014). Reagent and laboratory contamination can critically impact sequence-based microbiome analyses. BMC Biol..

[10] Vásquez A, Jakobsson T, Ahrné S, Forsum U, Molin G (2002). Vaginal *Lactobacillus* Flora of healthy Swedish women. J. Clin. Microbiol..

[11] Gibbs RS, Blanco JD, St Clair PJ, Castaneda YS (1982). Quantitative bacteriology of amniotic fluid from women with clinical intraamniotic infection at term. J. Infect. Dis..

[12] Jiménez E (2005). Isolation of commensal bacteria from umbilical cord blood of healthy neonates born by cesarean section. Curr. Microbiol..

[13] Hogue R, Graves M, Moler S, Janda JM (2007). Pink-pigmented non-fermentative gram-negative rods associated with human infections : a clinical and diagnostic challenge. Infection.

[14] Flynn AN, Lyndon CA, Church DL (2013). Identification by 16S rRNA gene sequencing of an Actinomyces hongkongensis isolate recovered from a patient with pelvic actinomycosis. J. Clin. Microbiol..

[15] Chang D-H, Rhee M-S, Kim B-C (2016). Dermabacter vaginalis sp. nov., isolated from human vaginal fluid. Int. J. Syst. Evol. Microbiol..

[16] McCormack WM (1977). Vaginal colonization with Corynebacterium vaginale (Haemophilus vaginalis). J. Infect. Dis..

[17] Holcombe LJ (2010). Pseudomonas aeruginosa secreted factors impair biofilm development in *Candida albicans*. Microbiology.

[18] Ligon JM (2000). Natural products with antifungal activity from Pseudomonas biocontrol bacteria. Pest Manag. Sci..

[19] Kim TK (2009). Heterogeneity of vaginal microbial communities within individuals. J Clin Microbiol..

[20] Yu RR (2009). A Chinese rhesus macaque (Macaca mulatta) model for vaginal *Lactobacillus* colonization and live microbicide development. J. Med. Primatol..

[21] Albert AYK (2015). A study of the vaginal microbiome in healthy Canadian women utilizing cpn60-based molecular profiling reveals distinct Gardnerella subgroup community state types. PLoS ONE.

[22] Pelzer ES (2012). Hormone-dependent bacterial growth, persistence and biofilm formation - a pilot study investigating human follicular fluid collected during IVF cycles. PLoS ONE.

[23] Pelzer ES (2013). TUNEL analysis of DNA fragmentation in mouse unfertilized oocytes: The effect of microorganisms within human follicular fluid collected during IVF cycles. J. Reprod. Immunol..

[24] Pelzer ES (2013). Microorganisms within human follicular fluid: effects on IVF. PLoS ONE.

[25] Mackie RI, Sghir A, Gaskins HR (1999). Developmental microbial ecology of the neonatal gastrointestinal tract. Am. J. Clin. Nutr..

[26] Matamoros S, Gras-Leguen C, Le Vacon F, Potel G, De La Cochetiere MF (2013). Development of intestinal microbiota in infants and its impact on health. Trends Microbiol..

[27] Bäckhed F (2015). Dynamics and stabilization of the human gut microbiome during the first year of life. Cell Host Microbe.

[28] Funkhouser LJ, Bordenstein SR (2013). Mom knows best: the Universality of maternal microbial transmission. PLoS Biol..

[29] Cicinelli E (2008). Chronic endometritis: correlation among hysteroscopic, histologic, and bacteriologic findings in a prospective trial with 2190 consecutive office hysteroscopies. Fertil. Steril..

[30] Walther-António MRS (2016). Potential contribution of the uterine microbiome in the development of endometrial cancer. Genome Med.

[31] Huang B, Fettweis JM, Brooks JP, Jefferson KK, Buck Ga (2014). The changing landscape of the vaginal microbiome. Clin. Lab. Med..

[32] Wang J, Jia H (2016). Metagenome-wide association studies: fine-mining the microbiome. Nat. Rev. Microbiol..

[33] Qin J (2012). A metagenome-wide association study of gut microbiota in type 2 diabetes. Nature.

[34] Schloss PD (2009). Introducing mothur: open-source, platform-independent, community-supported software for describing and comparing microbial communities. Appl. Environ. Microbiol..

[35] Caporaso JG (2010). QIIME allows analysis of high-throughput community sequencing data. Nat. Methods.

[36] Dufrêne M, Legendre P (1997). Species assemblages and indicator species:the need for a flexible asymmetrical approach. Ecol. Monogr..

[37] Anderson MJ (2001). A new method for non-parametric multivariate analysis of variance. Aust. Ecol..

[38] Feng Q (2015). Gut microbiome development along the colorectal adenoma–carcinoma sequence. Nat. Commun..

[39] Zapala MA, Schork NJ (2006). Multivariate regression analysis of distance matrices for testing associations between gene expression patterns and related variables. Proc. Natl Acad. Sci. USA.

[40] Subramanian S (2014). Persistent gut microbiota immaturity in malnourished Bangladeshi children. Nature.

[41] Zhang X (2015). The oral and gut microbiomes are perturbed in rheumatoid arthritis and partly normalized after treatment. Nat. Med..

[42] Langille MGI (2013). Predictive functional profiling of microbial communities using 16S rRNA marker gene sequences. Nat. Biotechnol..

[43] Patil KR, Nielsen J (2005). Uncovering transcriptional regulation of metabolism by using metabolic network topology. Proc. Natl Acad. Sci. USA.

[44] De Backer E (2007). Quantitative determination by real-time PCR of four vaginal *Lactobacillus* species, *Gardnerella vaginalis* and *Atopobium vaginae* indicates an inverse relationship between *L. gasseri* and *L. iners*. BMC Microbiol..

[45] Augustinos AA (2015). Exploitation of the medfly gut microbiota for the enhancement of sterile insect technique: Use of Enterobacter sp. in larval diet-based probiotic applications. PLoS ONE.

