# Supplementary material for: The microbiota continuum along the female reproductive tract and its relation to uterine-related diseases
Source: Nat Commun. 2017 Oct 17;8:875. doi: 10.1038/s41467-017-00901-0 (PMC5645390; doi:10.1038/s41467-017-00901-0)
Supplement: Supplementary file 1 — Supplementary Information [file 41467_2017_901_MOESM1_ESM.pdf]

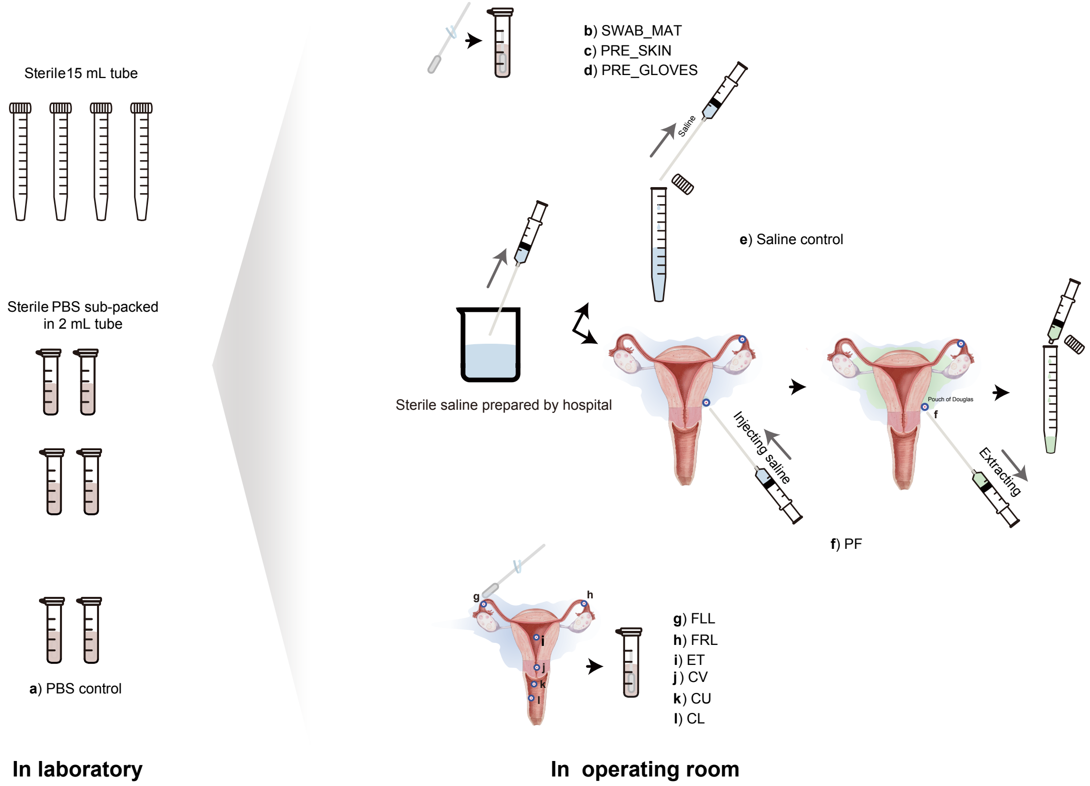

Supplementary Figure 1: Sample collection. (a) Sterile PBS in 2 mL tube prepared in the laboratory. **(b)** Dry sterile swabs material (SWAB_MAT). The preoperative skin area was sterilizing with Andoful, then dry sterile swabs rubbed on **(c)** patient's preoperative skin area (PRE_SKIN) and **(d)** surgeon's gloved fingers (PRE_GLOVES). **(e)** Sterile physiological saline prepared by hospital, 10 mL physiological saline was transferred to a sterile tube. **(f)** After injection of sterile physiological saline into peritoneal cavity, the PF was extracted with the injector, and transfered to a sterile tube. Dry sterile swabs rubbed on different sites of reproductive tract **(g**, FLL; **h**, FRL; **i**, ET; **j**, CV; **k**, CU; **l**, CL**)**. Swabs were then transferred to tubes with sterile PBS **(b, c, d, g, h, i, j, k, l)**. Samples (**f, g, h, i, j, k, l**) were collected from the initial cohort of 95 reproductive-age women. Samples (**b, c, d, e, f, g, h, i**) were collected from the additional cohort of 15 reproductive-age women.

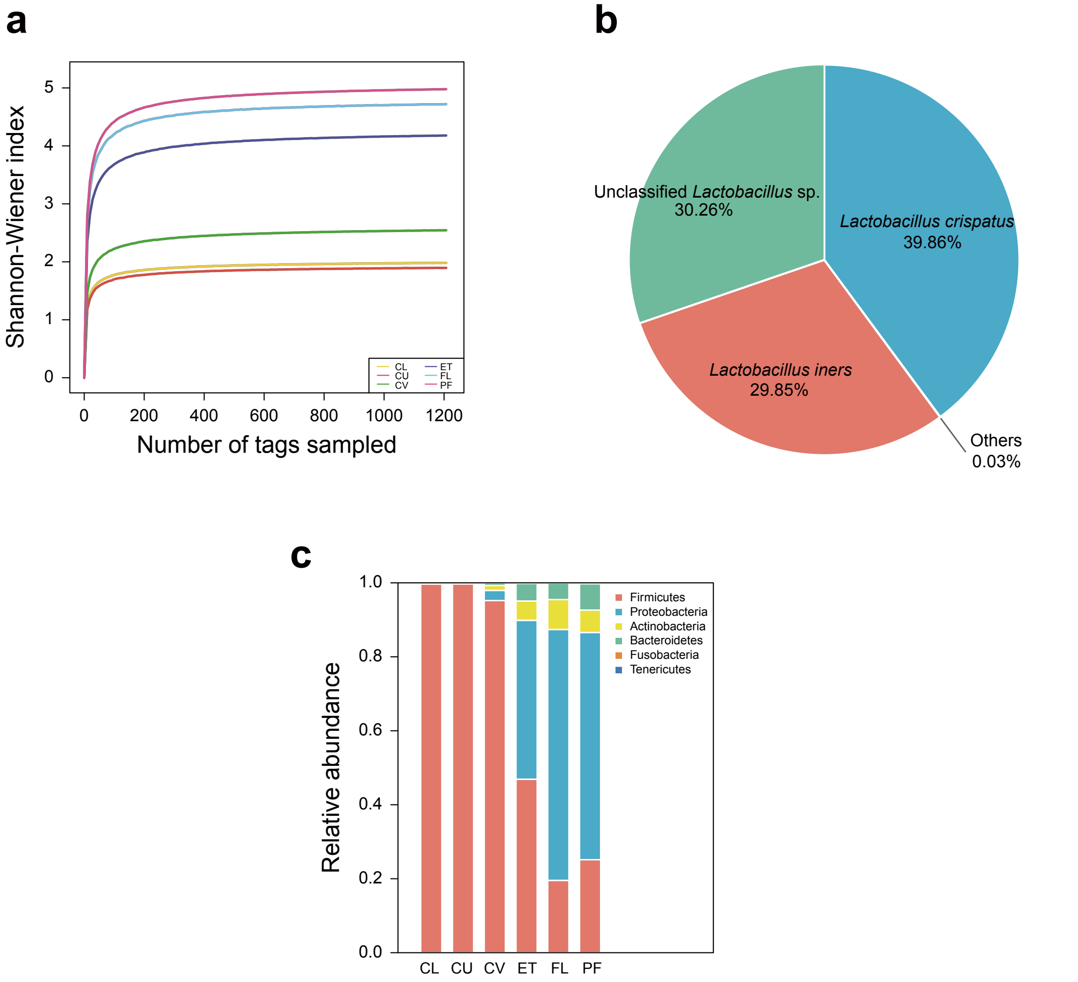

Supplementary Figure 2: Diversity and composition of the vagino-uterine microbiota. Samples derive from the study cohort of 95 reproductive-age women (**Supplementary Data 1**). **(a)** α-diversity of the samples from each body site. Shannon index was calculated from the relative abundance of all OTUs in each sample. **(b)** Species composition of the vaginal microbiota. Pie chart for the microbial species at the CL, according to their median relative abundances. Species that took up less than 1% of the microbiota were labeled together as 'Others'. **(c)** Major phyla in the female reproductive tract. The median of the relative abundance of each phylum in the samples was plotted.

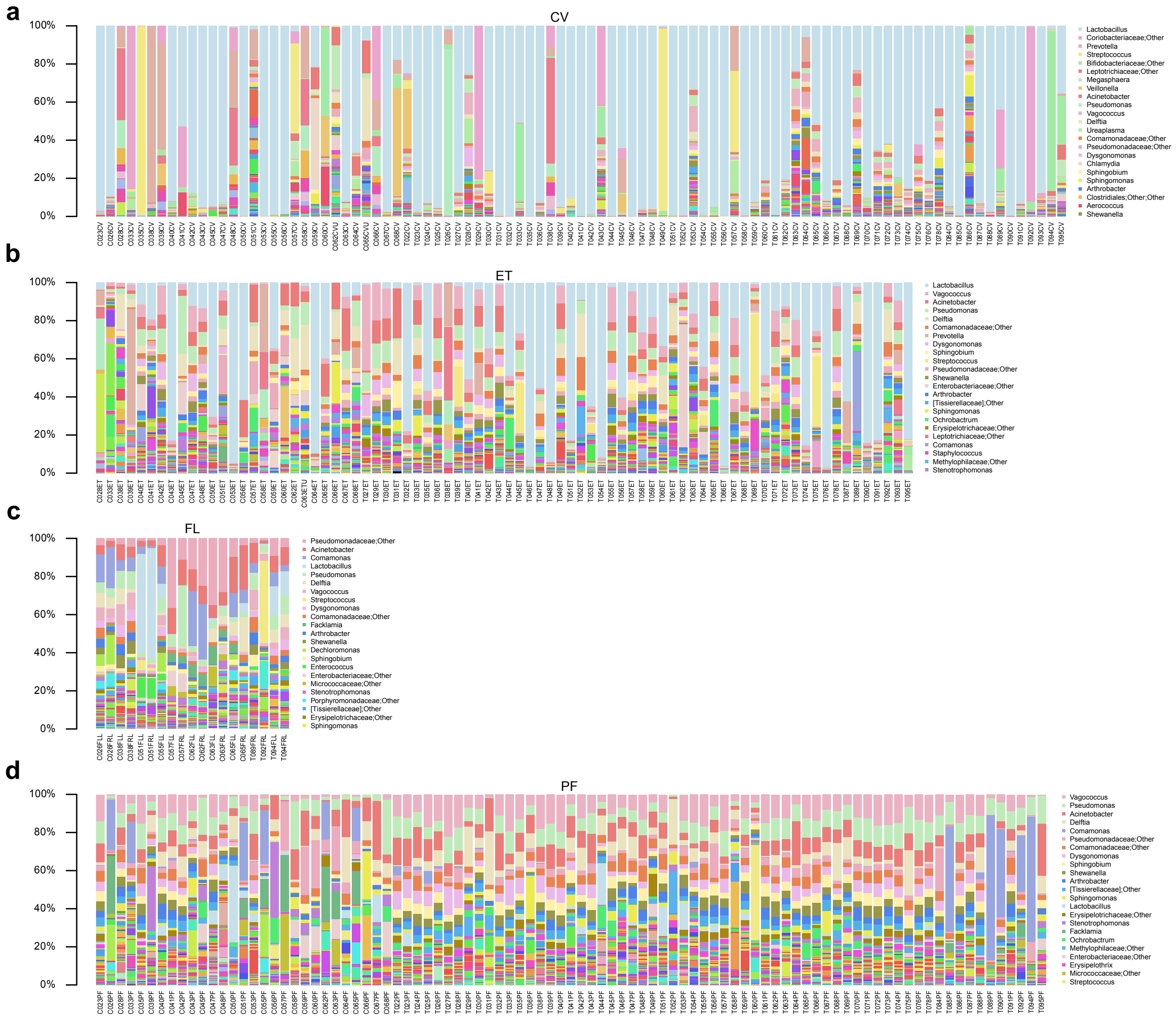

Supplementary Figure 3: Cumulative bar charts of the main taxa at genus and family levels present at each body site. (a) CV. **(b)** ET. **(c)** FL. **(d)** PF. Each column represents an individual sample collected in the study cohort of 95 reproductive-age women.

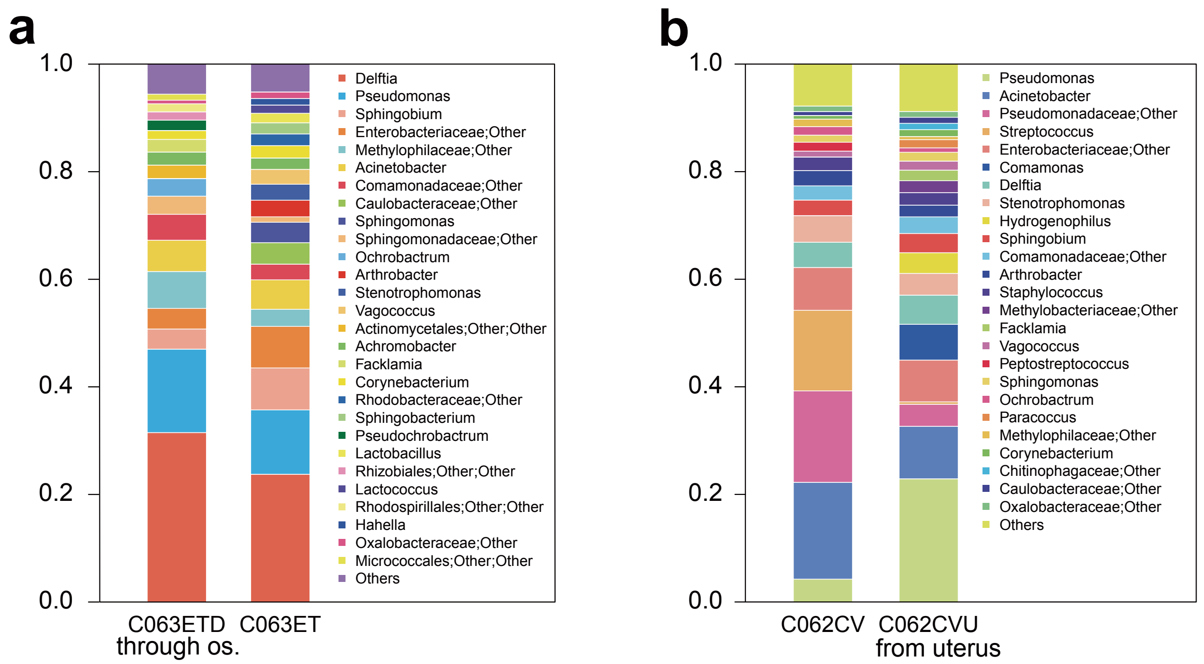

C063	Pearson correlation		n (N)
	OTU	Genus	
CL-ETD	-6.39E-03	9.30E-03	7 (213)
ETD-ET	0.879	0.915	58 (155)
CL-ET	-8.98E-03	4.68E-02	3 (222)

C062	Pearson correlation		n (N)
	OTU	Genus	
CL-CV	0.350	0.473	59 (285)
CV-CVU	0.382	0.515	91 (249)
CL-CVU	4.83E-03	3.99E-03	40 (280)

Supplementary Figure 4: The relative abundance of each genus in samples taken via two sampling routes from the same individual. (a) ET samples taken directly from the uterus (C063ET) and through the cervical os (C063ETD). C063ET and C063ETD were taken on the same day. Hyphen indicates correlation between samples from two different body sites from the same individual, such as CL-ETD, the correlation between CL sample and ETD sample from C063. (**b**) CV samples taken from the cervical os (C062CV) and through the uterine end (C062CVU). C062CVU was taken 4 days after C062CV. n indicates number of common OTUs, N indicates total number of OTUs. Samples derive from the study cohort of 95 reproductive-age women.

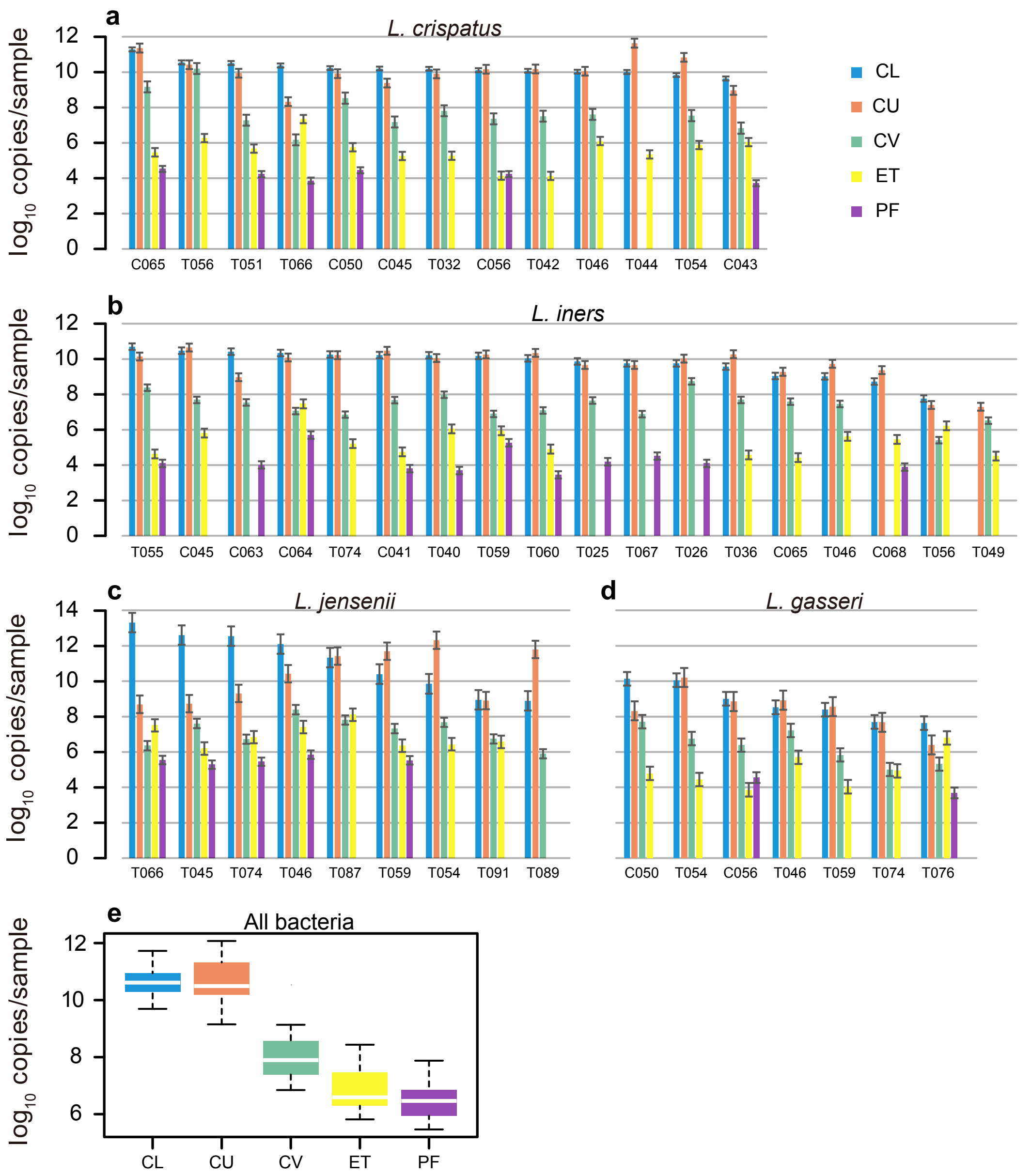

Supplementary Figure 5: The presence and average concentrations of the dominant *Lactobacillus* species at each body site determined by real-time qPCR. (a) *Lactobacillus crispatus*. **(b)** *Lactobacillus iners*. **(c)** *Lactobacillus jensenii*. **(d)** *Lactobacillus gasseri*. Error bar represents the standard deviation of three replicate measurements. **(e)** The median total bacterial counts at each site, calculated based on the copy number of *L. iners* from qPCR divided by the corresponding relative abundance in the sample according to 16S rRNA gene sequencing. Boxes denote the interquartile range (IQR) between the first and third quartiles (25th and 75th percentiles, respectively), and the line inside the boxes denote the median. The whiskers denote the lowest and highest values within 1.5 times the IQR from the first and third quartiles, respectively. The samples originate from the study cohort of 95 women.

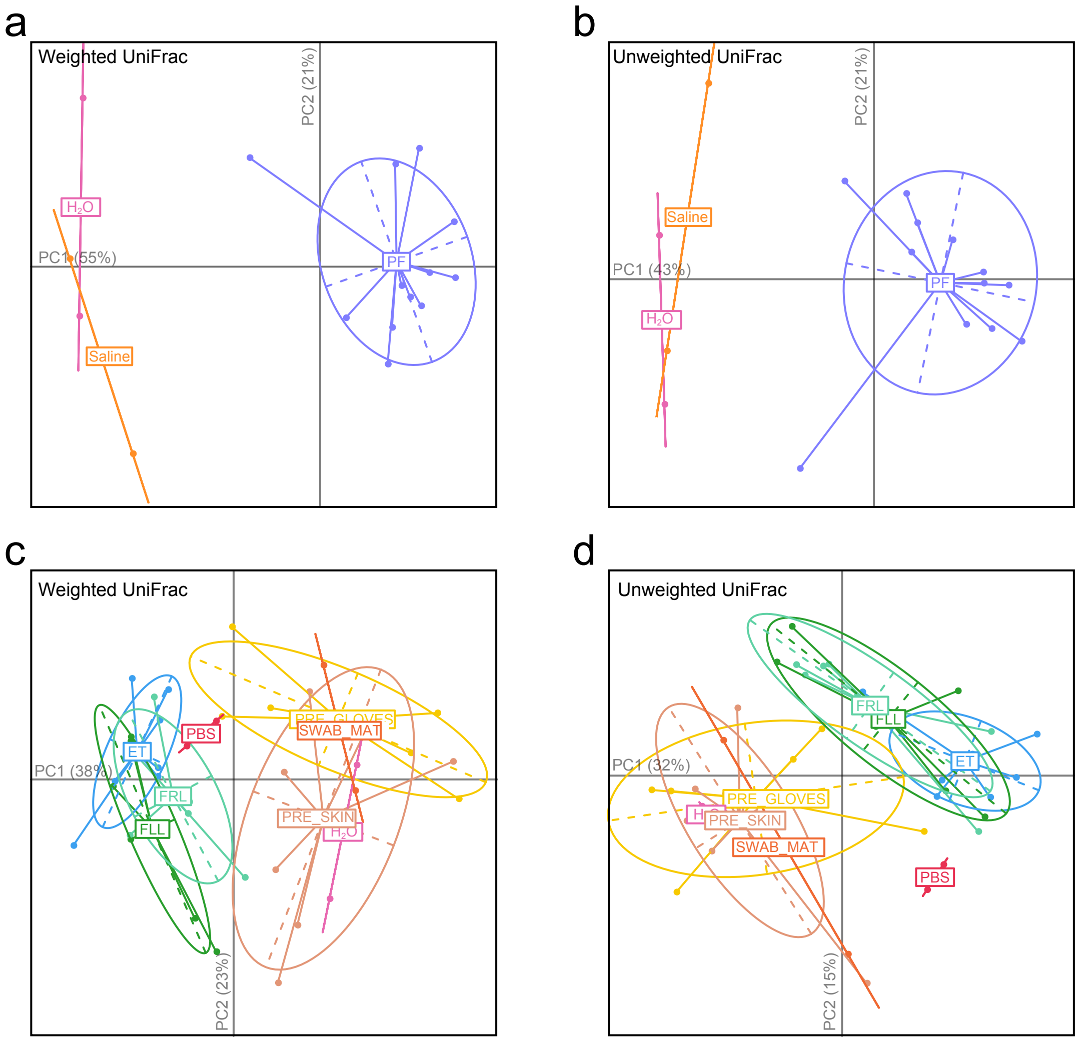

Supplementary Figure 6: PCoA comparing upper reproductive tract samples with their respective diluent controls based on weighted (a, c) and unweighted (b, d) UniFrac distances. **(a, b)** PF and sterile physiological saline, saline was the respective controls of PF prepared by hospital. **(c, d)** FL, ET, their diluent negative controls and PBS. SWAB_MAT, dry sterile swabs material; PRE_GLOVES, dry sterile swabs rubbed on surgeon's gloved fingers; PRE_SKIN, dry sterile swabs rubbed on patient's preoperative skin area; H_2O, ultrapure water. These samples originate from the cohort of 15 additional reproductive-age women.

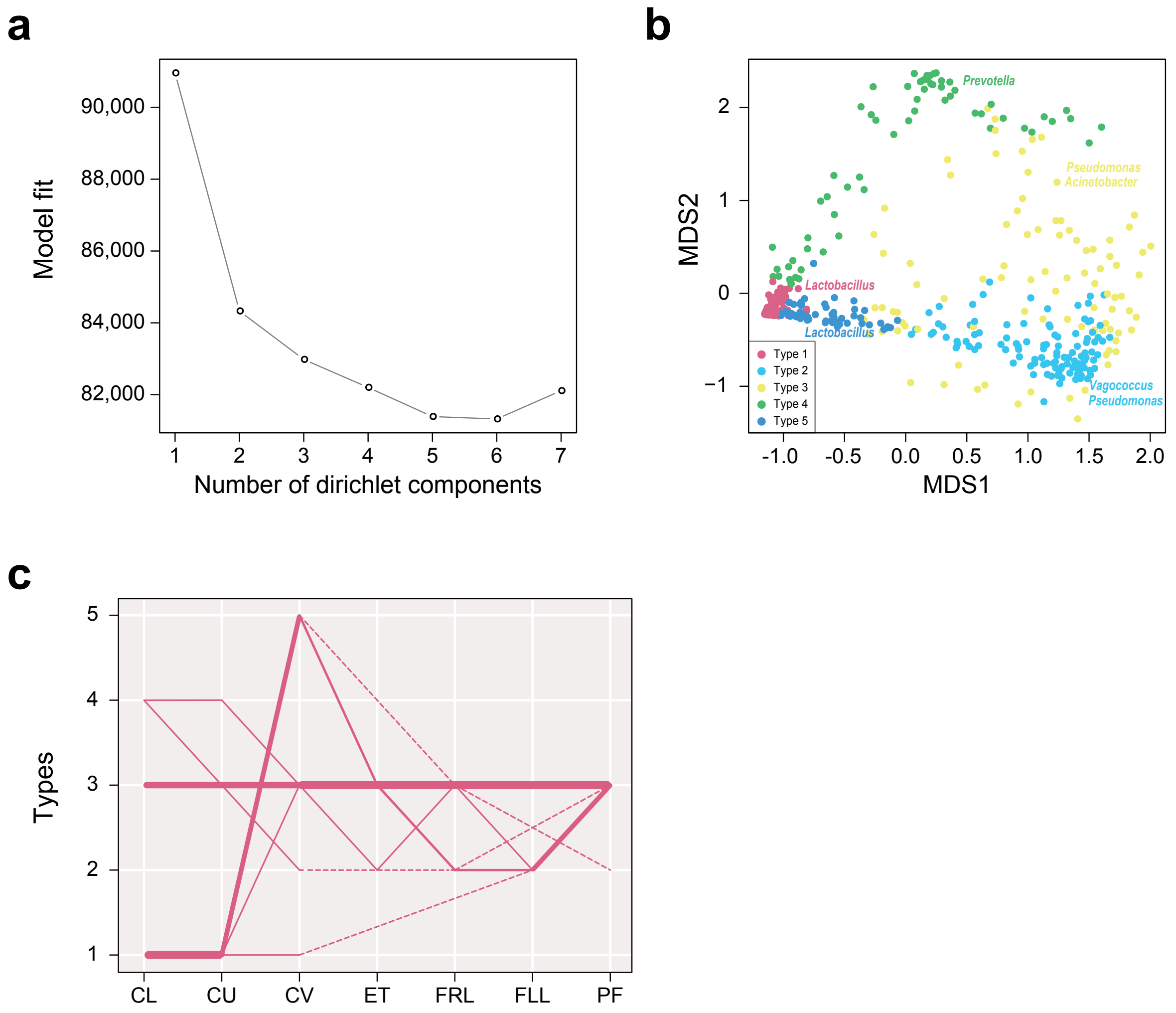

Supplementary Figure 7: Difference in community types of the vagino-uterine microbiota. (a) Fitting to the DMM model indicates optimal classification into five or six community types. **(b)** Plot for non-metric dimensional scaling (NMDS) ordination of Jensen–Shannon divergence values between all samples using DMM. **(c)** Community types connected between neighboring sites, for individuals with fallopian tube samples (n = 1~11).

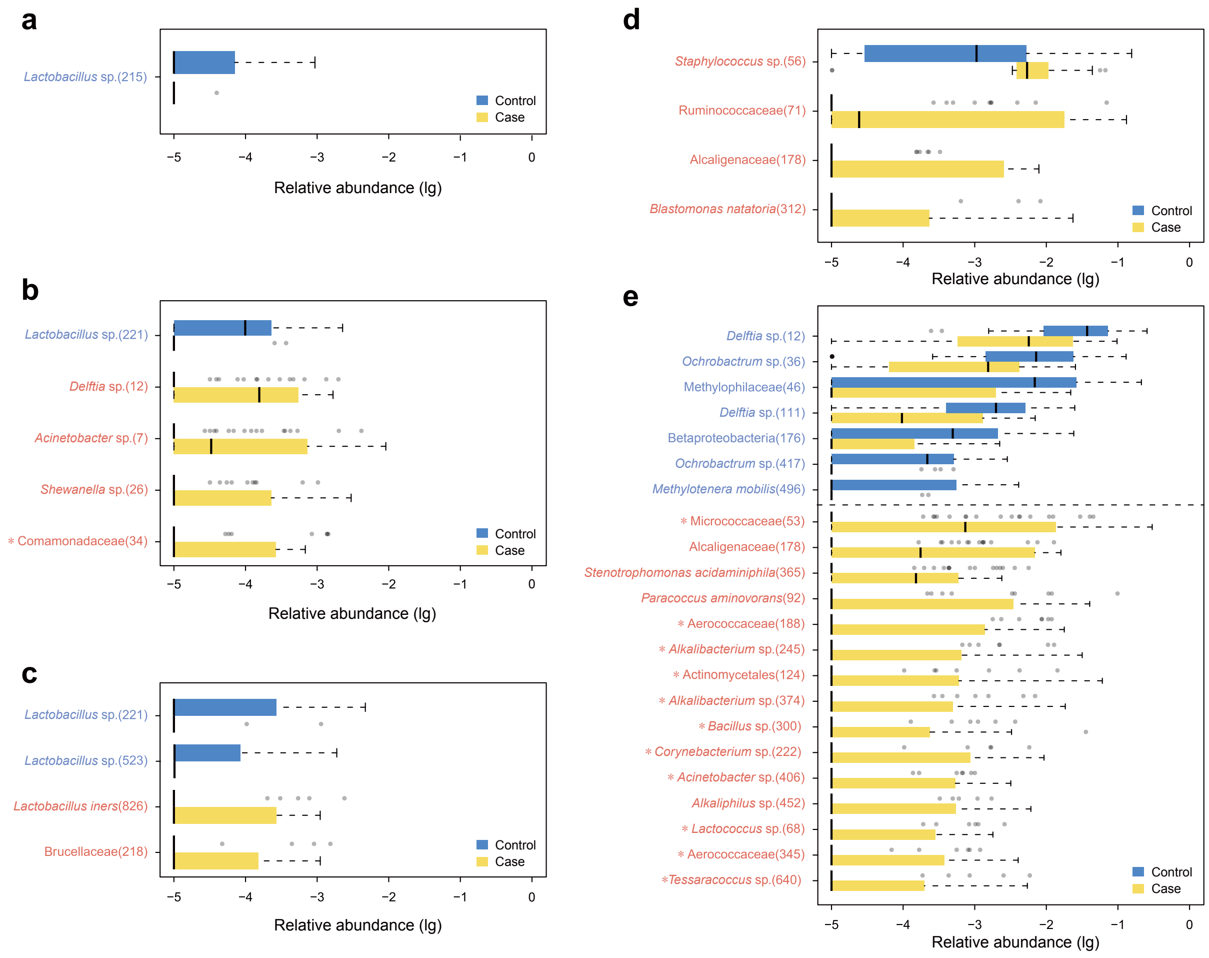

Supplementary Figure 8: OTUs differentially enriched between subjects with and without hysteromyoma (n = 20 cases, 75 controls). (a) CL. **(b)** CU. **(c)** CV. **(d)** ET. **(e)** PF. $P < 0.05$, $q < 0.05$, Wilcoxon-rank sum test. OTU identification numbers are shown in parenthesis. Asterisks indicate possible influence from menstrual cycle, i.e. the relative abundance of an OTU correlates with days in the menstrual cycle (Spearman's correlation coefficient > 0.3 or < -0.3, $q < 0.05$). Boxes denote the interquartile range (IQR) between the first and third quartiles (25th and 75th percentiles, respectively), and the line inside the boxes denote the median. The whiskers denote the lowest and highest values within 1.5 times the IQR from the first and third quartiles, respectively.

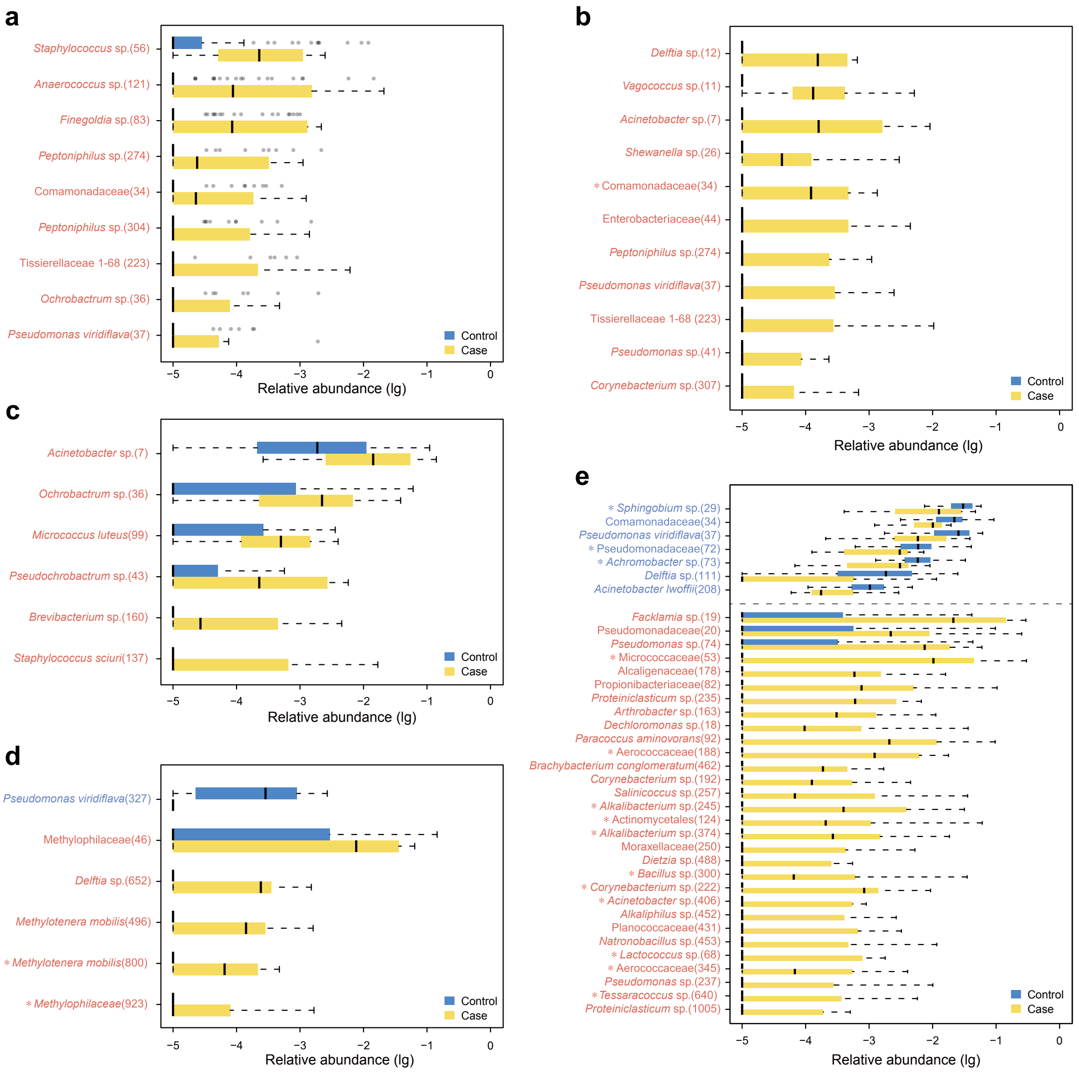

Supplementary Figure 9: OTUs differentially enriched between subjects with and without adenomyosis (n = 14 cases, 81 controls). (a) CL. **(b)** CU. **(c)** CV. **(d)** ET. **(e)** PF. $P < 0.05$, $q < 0.05$, Wilcoxon-rank sum test. OTU identification numbers are shown in parenthesis. Asterisks indicate possible influence from menstrual cycle, i.e. the relative abundance of an OTU correlates with days in the menstrual cycle (Spearman's correlation coefficient > 0.3 or < -0.3, $q < 0.05$). Boxes denote the interquartile range (IQR) between the first and third quartiles (25th and 75th percentiles, respectively), and the line inside the boxes denote the median. The whiskers denote the lowest and highest values within 1.5 times the IQR from the first and third quartiles, respectively.

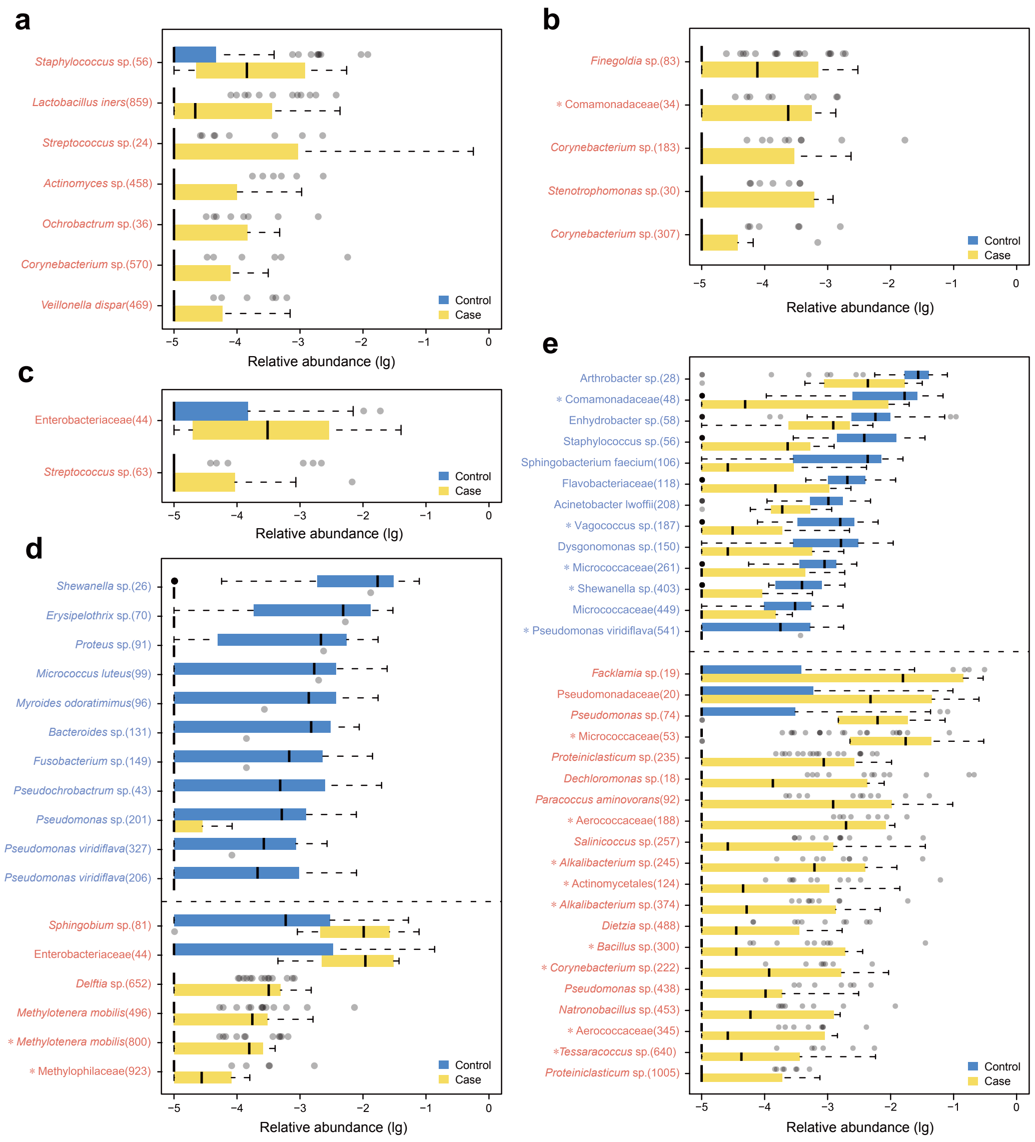

Supplementary Figure 10: OTUs differentially enriched between subjects with and without anemia (n = 11 cases, 84 controls). (a) CL. **(b)** CU. **(c)** CV. **(d)** ET. **(e)** PF. $P < 0.05$, $q < 0.05$, Wilcoxon-rank sum test. OTU identification numbers are shown in parenthesis. Asterisks indicate possible influence from menstrual cycle, i.e. the relative abundance of an OTU correlates with days in the menstrual cycle (Spearman's correlation coefficient > 0.3 or < -0.3, $q < 0.05$). Boxes denote the interquartile range (IQR) between the first and third quartiles (25th and 75th percentiles, respectively), and the line inside the boxes denote the median. The whiskers denote the lowest and highest values within 1.5 times the IQR from the first and third quartiles, respectively.

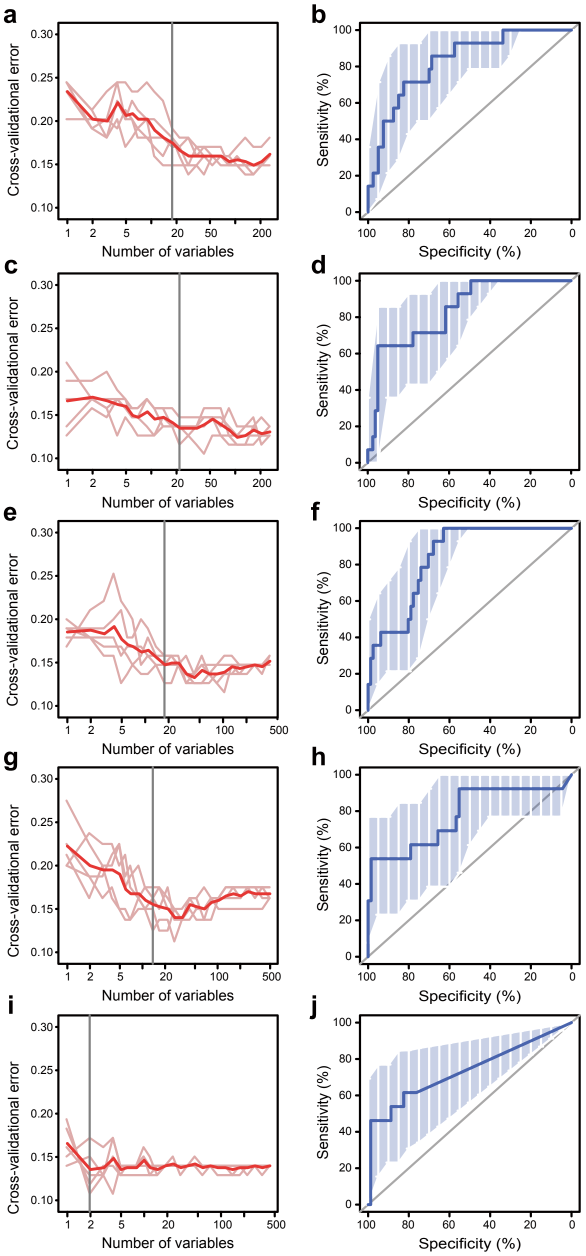

Supplementary Figure 11: Microbiota-based classification of adenomyosis. (a, c, e, g, i) Distribution of 5 trials of 10-fold cross-validation error in random forest classification of samples with and without adenomyosis as the number of OTUs increases (**a**, CL; **c**, CU; **e**, CV; **g**, ET; **i**, PF). The model was trained using relative abundance of the OTUs (present in at least 10% of the samples) in the samples (n = 14 with, 81 without adenomyosis). The red curve indicates average of the five trials (pink lines). The grey line marks the number of OTUs in the optimal set. **(b**, **d**, **f**, **h**, **j)** ROC for the cross-validated sample set (**b**, CL; **d**, CU; **f**, CV; **h**, ET; **j**, PF). The AUC is 0.8668, 0.8404, 0.8369, 0.7767 and 0.7341, respectively. The 95% confidence intervals (CI) are shown as shaded areas. The diagonal lines mark an AUC of 0.5 (i.e., random classification).

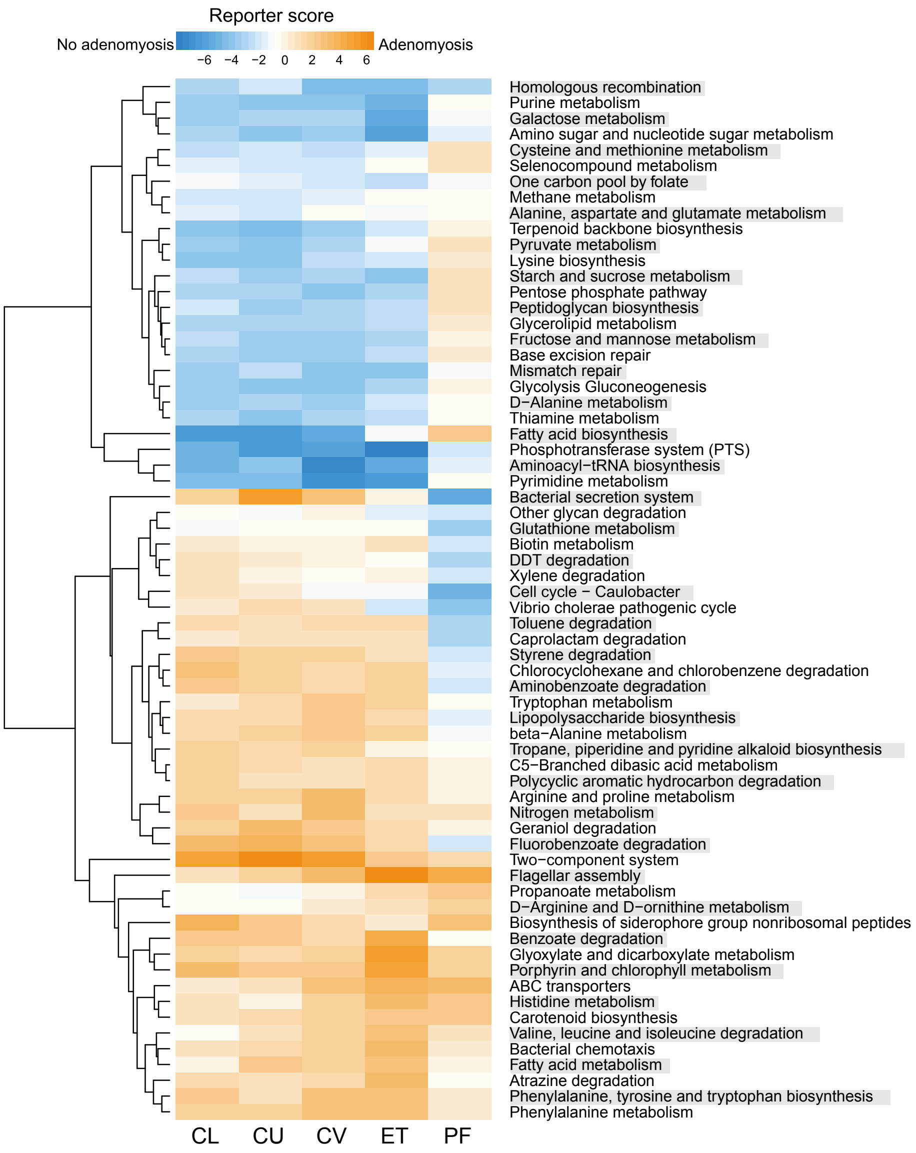

Supplementary Figure 12: Functions influenced by adenomyosis (n = 14 cases, 81 controls). KEGG pathways enriched in the samples with and without adenomyosis (orange versus blue, reporter score > 1.96 or < -1.96) and present in all the sites were plotted as a heatmap. The pathways were arranged by unsupervised hierarchical clustering.

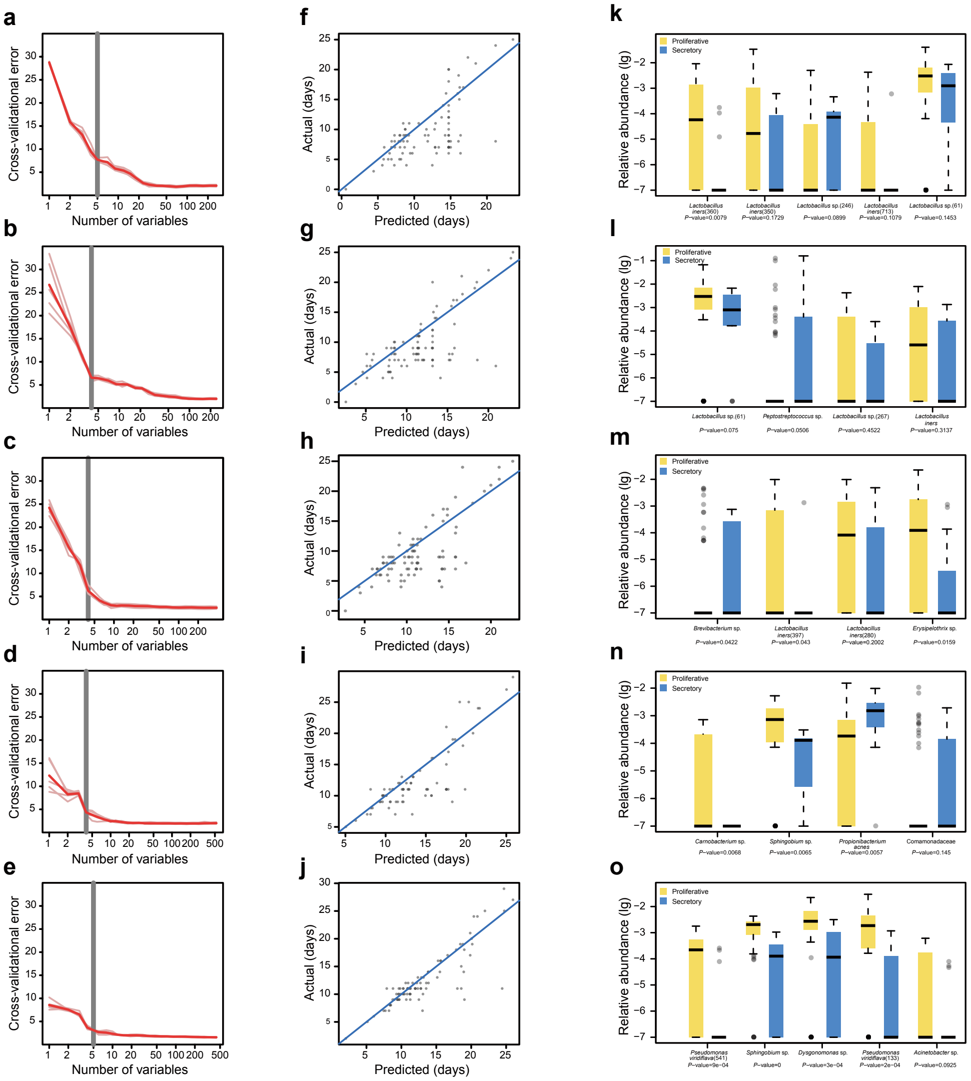

Supplementary Figure 13: Microbiota-based classification of days in the menstrual cycle. (a, b, c, d, e) Distribution of 5 trials of 10-fold cross-validation error in random forest classification of samples into days in the menstrual cycle (continuous variable) as the number of OTUs increases (**a**, CL; **b**, CU; **c**, CV; **d**, ET; **e**, PF). The model was trained using relative abundance of the OTUs (present in at least 10% of the samples) in the samples (n = 95). The red curve indicates average of the five trials (pink lines). The grey line marks the number of OTUs in the optimal set. **(f, g, h, i, j)** Actual and predicted days in the menstrual cycle according to the random forest models (**f**, CL; **g**, CU; **h**, CV; **i**, ET; **j**, PF). Adjusted R^2 = 0.5404, 0.4843, 0.4169, 0.6563 and 0.7375, respectively. **(k, l, m, n, o)** Relative abundances of OTUs selected by the random forest models (**k**, CL; **l**, CU; **m**, CV; **n**, ET; **o**, PF). P-values from Wilcoxon-rank sum tests. Boxes denote the interquartile range (IQR) between the first and third quartiles (25th and 75th percentiles, respectively), and the line inside the boxes denote the median. The whiskers denote the lowest and highest values within 1.5 times the IQR from the first and third quartiles, respectively.

Supplementary Table 1 The Ct value of samples and negative controls in four Lactobacillus species at ET and PF.

**Species**	**Samples**						**Negative controls**		
	**ET (Ct[a]<30)**		**ET(Ct>30)**	**PF(Ct<30)**		**PF(Ct>30)**	**PBS (Ct)**	**Saline (Ct)**	**H_2O (Ct)**
	**sample number**	**Ct median**	**sample number**	**sample number**	**Ct median**	**sample number**			
L.iners	14	23.99	1	11	28.08	6	30.95	30.57	31.04
L. crispatus	13	24.18	0	6	28.67	6	32.37	33.55	33.55
L. gasseri	7	26.26	0	2	28.53	5	30.64	30.97	31.02
L. jensenii	8	23.25	1	6	27.10	4	32.31	33.29	32.96

[a] Ct values are shown as average of triplicate measures.

Supplementary Table 2 Pearson correlation to samples collected in the cohort of 15 additional women and treated by different protocols

Different processes	Samples	ID_A[a]_B[b]_C[c]	Pearson correlation	
			OTU	Genus
DNA extraction rounds	C001PF	2_1_2 vs 1_1_2	0.994772	0.99463
	C002PF	1_1_1 vs 2_1_1	0.943833	0.948424
	T001PF	1_1_1 vs 2_1_1	0.973382	0.975381
	T003PF	1_1_2 vs 2_1_2	0.843397	0.823239
Chip	T002PF	1_1_1 vs 2_1_2	0.966119	0.968816
	C001PF	1_1_1 vs 1_1_2	0.956075	0.959856
	P006PF	1_1_1 vs 1_1_2	0.996552	0.996289
Dilution and chip	P001FLL	1_1_1 vs 1_0_2	0.994538	0.993914
	P002PF	1_1_1 vs 1_0_2	0.946928	0.958866
	P005PF	1_1_1 vs 1_0_2	0.989396	0.992172
	P008PF	1_1_1 vs 1_0_2	0.937114	0.94679

[a] A indicates DNA was extracted at two time points/rounds, 1: first, 2: second

[b] B indicates that DNA template was diluted or not before PCR, 0: diluted. 1: without dilution

[c] C indicates that two chips were used, 1: first chip. 2: second chip

Supplementary Table 3 Identification of cultured microbial isolates from peritoneal fluid of the 15 additional women by sequencing of partial 16S gene.

No.	Bacteria isolated	GenBank accession	Seqmatch score Identity	sample ID	aerobically culture	anaerobically culture	Observed in 16S rRNA sequencing in validation study	Supported by previous cultivation
1	*Lactobacillus brevis*	KX959612	100%	C002, T002		√	√	vaginal fluid[10] , amniotic fluid[11]
2	*Staphylococcus epidermidis*	KX959613	100%	T003	√	√	√	amniotic fluid[11], umbilical cord blood[12]
3	*Staphylococcus* sp.	KX959614	100%	T003	√	√	√	amniotic fluid[11], umbilical cord blood[12]
4	*Actinomyces radingae*	KX959617	99%	P007		√	√	pelvic fluid[13]
5	*Corynebacterium tuberculostearicum*	KX959619	100%	P007	√	√	√	vaginal fluid[14]
6	*Propionibacterium avidum*	KX959616	100%	P007	√	√	√	amniotic fluid[11], umbilical cord blood[12]
7	*Dermabacter hominis*	KX959618	99%	P007		√	√	vaginal fluid[15]
8	*Roseomonas* sp.	KX959615	100%	P004	√		√	cervix[16]

Supplementary Table 4 Comparison with the chronic endometriosis study by Cicinelli *et al*.

	**Cicinelli *et al*. (Culture for bacteria, yeast, PCR for Chlamydia)**				**This study (16S)**				
	Chronic endometritis at hysteroscopy		Control group						
**Infectious agent**	Vagina (438)	Endometriuma (438)	Vagina (100)	Endometrium (100)	CL (94)	CU (95)	CV (95)	ET (80)	PF (93)
Escherichia coli	38 (8.7%)	50 (11.4%)	15 (15.0%)	1 (1.0%)	0	0	0	0	0
Streptococci	80 (18.3%)	122 (27.9%)	16 (16.0%)	2 (2.0%)	43 (45.7%)	43 (45.3%)	59 (62.1%)	49 (61.3%)	56 (60.2%)
Staphylococci	0	20 (4.6%)	1 (1.0%)	0	33 (35.1%)	31 (32.6%)	62 (65.3%)	63 (78.8%)	81 (87.1%)
Enterococcus faecalis	22 (5.0%)	62 (14.2%)	7 (7.0%)	1 (1.0%)	0	0	0	0	0
Chlamydia	2 (0.5%)	12 (2.7%)	0	0	3 (3.2%)	1 (1.1%)	4 (4.2%)	4 (5.0%)	0
Ureaplasma	86 (19.6)	44 (10.0%)	5 (5.0%)	1 (1.0%)	52 (55.3%)	47 (49.5%)	43 (45.3%)	20 (25.0%)	2 (2.2%)
Yeast	26 (5.9%)	10 (2.3%)	10 (10.0%)	0	NA	NA	NA	NA	NA

Supplementary Table 5 Comparison with the endometrial cancer study by Walther-António *et al*.

		**Walther-António *et al*.**			**This study**
**Clinical conditions**		Benign (n = 10)	Cancer (n = 17)	Hyperplasia (n = 4)	Benign (n=110)
**Age (years) – Median, IQR**		44.5 (42.5–52.5)	64 (58–71)	54 (50.75–62.5)	31.5 (27-35)
**Ethnicity**		Caucasian			Chinese
**Menopausal status**		14 Pre/Peri, 17 Post			110 Pre
Dominate bacteria	Vagina and cervix	*Lactobacillus, Prevotella*			*Lactobacillus*
	Uterus	*Shigella, Parabacteroides, Barnesiella, Bacteroides*			*Lactobacillus, Pseudomonas, Acinetobacter, Vagococcus*
	Fallopian tube	*Shigella, Bacteroides*			*Pseudomonas, Acinetobacter, Comamonas*
	Ovary	*Shigella, Bacteroides*			NA
Occurrence of cancer-associated bacteria in vagina and cervix	*Atopobium sp.*	4/10	14/15	1/4	24/95, mean abundance ~10^{-4}
	Porphyromonas sp.	2/10	12/15	1/4	23/95, mean abundance ~ 10^{-4}
