# Supplementary material for: The microbiota continuum along the female reproductive tract and its relation to uterine-related diseases
Source: Nat Commun. 2017 Oct 17;8:875. doi: 10.1038/s41467-017-00901-0 (PMC5645390; doi:10.1038/s41467-017-00901-0)
Supplement: Supplementary file 2 — Description of Additional Supplementary Files [file 41467_2017_901_MOESM2_ESM.pdf]

Description of Additional Supplementary Files

File Name: Supplementary Data 1
Description: Phenotypic information for the initial study cohort of 95 women.

File Name: Supplementary Data 2
Description: Sequencing and annotation for the study cohort of 95 women.

File Name: Supplementary Data 3
Description: List of OTUs. * Experimenally determined.

File Name: Supplementary Data 4
Description: Sequencing and annotation for the cohort of 15 additional women.

File Name: Supplementary Data 5
Description: Sequencing and annotation for the cohort of 15 additional women in different protocols.

File Name: Supplementary Data 6
Description: Signature OTUs for each site of the vagino-uterine microbiota. Based on IndVal, a well-established method in ecology considering both the occurrence and abundance of species.

File Name: Supplementary Data 7
Description: Community types of the vagino-uterine microbiota for all samples from the study cohort of 95 women.

File Name: Supplementary Data 8
Description: PERMANOVA for the influence of phenotypes on the microbiota. 9999 permutations, Bray-Curtis distance. Phenotypes with $p < 0.05$ in at least one site are shown. FDR control using the Benjamini-Hochberg procedure.

File Name: Supplementary Data 9
Description: PERMANOVA for the influence of phenotypes on the microbiota. 9999 permutations, unweighted UniFrac distance. Phenotypes with $p < 0.05$ in at least one site are shown. FDR control using the Benjamini-Hochberg procedure.

File Name: Supplementary Data 10
Description: OTUs included in the random forest classifiers for adenomyosis.

File Name: Supplementary Data 11
Description: OTUs included in the random forest classifiers for infertility and infertility due to endometriosis.
